# New‐Onset Type 2 Diabetes Mellitus and Cancer Risk: A Matched Cohort Study in China Kadoorie Biobank

**DOI:** 10.1002/ijc.70638

**Published:** 2026-07-09

**Authors:** Mengying Wang, Owen Tipping, Bowen Liu, Derrick A. Bennett, Baiyong Shen, Siyi Zou, Shulin Zhao, Richard M. Martin, Matthew Sperrin, Andrew G. Renehan

**Affiliations:** ^1^ Division of Cancer Sciences, School of Medical Sciences, Faculty of Biology, Medicine and Health University of Manchester Manchester UK; ^2^ Clinical Trial Service Unit and Epidemiological Studies Unit, Nuffield Department of Population Health University of Oxford Oxford UK; ^3^ Department of General Surgery Pancreatic Disease Centre, Ruijin Hospital, Shanghai Jiao Tong University School of Medicine Shanghai China; ^4^ Research Institute of Pancreatic Diseases, Shanghai Key Laboratory of Translational Research for Pancreatic Neoplasms, Shanghai Jiao Tong University School of Medicine Shanghai China; ^5^ State Key Laboratory of Oncogenes and Related Genes, Institute of Translational Medicine, Shanghai Jiao Tong University Shanghai China; ^6^ Department of Population Health Sciences, Bristol Medical School University of Bristol Bristol UK; ^7^ National Institute for Health Research (NIHR) Bristol Biomedical Research Centre Bristol UK; ^8^ Centre for Health Informatics, Division of Informatics, Imaging and Data Sciences, School of Health Sciences, Faculty of Biology, Medicine and Health University of Manchester Manchester UK; ^9^ NIHR Manchester Biomedical Research Centre Manchester UK

**Keywords:** cancer, China Kadoorie biobank, longitudinal matching, Mendelian randomisation, type 2 diabetes

## Abstract

The prevalence of type 2 diabetes mellitus (T2DM) is rising rapidly in China and is linked to increased cancer risk, but causality remains unclear due to biases. We examined the causal effect of T2DM on cancer risk using bias‐minimizing methods. We conducted a matched cohort study within China Kadoorie Biobank (median follow‐up 49 months in men and 53 months in women). To minimize bias, we included only new‐onset T2DM, applied sequential longitudinal matching, used pre‐diagnosis BMI, and accounted for detection time bias. Stratified Cox models estimated sex‐specific time‐split hazard ratios (tsHRs). Results were triangulated with two‐sample Mendelian Randomization (MR) in East Asians. After 1:3 matching, 8657 men and 13,680 women with T2DM were matched to unexposed individuals. T2DM was associated with increased risk of total cancers in men (tsHR 1.57, 95% CI 1.38–1.78) and women (tsHR 1.29, 95% CI 1.14–1.46). Site‐specifically, T2DM was associated with liver cancer (men: 68 cases, tsHR 2.12, 95% CI 1.42–3.15; women: 43 cases, tsHR 2.39, 95% CI 1.42–4.04) and pancreatic cancer (men: 36 cases, tsHR 2.57, 95% CI 1.35–4.92; women: 33 cases, tsHR 3.95, 95% CI 1.92–8.13). No significant associations were observed for colorectal, lung, stomach, or breast cancers. In multivariable MR, genetic liability to T2DM was associated with pancreatic cancer after adjusting for BMI (OR 1.07, 95% CI 1.01–1.14, p = 0.03). In this large Chinese population, we found evidence for causal associations between T2DM and pancreatic cancer only, whereas evidence for other cancers was weak or discordant.

## Abstract (highlights)

What's new?

Type 2 diabetes mellitus is associated with increased cancer risk, but causality remains unclear. This matched cohort study using the China Kadoorie Biobank suggests that, after minimizing key biases, new‐onset type 2 diabetes mellitus is associated with higher risk of all cancers and, in particular, cancers of the liver and pancreas in Chinese adults. Mendelian Randomization supported a causal effect of genetic liability to type 2 diabetes mellitus on pancreatic cancer, whereas evidence for other sites was weak. Cancer prevention strategies should not treat type 2 diabetes mellitus as a broad carcinogenic exposure but instead focus on modifiable upstream determinants.

## Graphical abstract

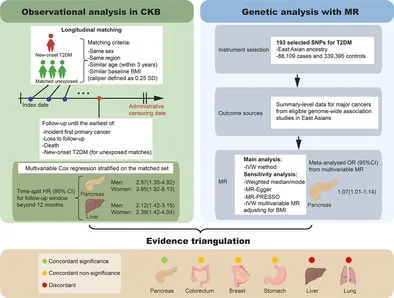

**Abbreviations**
BBJ: Biobank Japan
BMI: body mass index
CI: confidence interval
CKB: China Kadoorie Biobank
GWAS: genome‐wide association study
HBV: hepatitis B virus
HCV: hepatitis C virus
IARC: International Agency for Research on Cancer
ICD‐10: International Classification of Diseases 10th Revision
IGF: insulin‐like growth factor
IVW: inverse‐variance weighted
JaPAN: Japan Pancreatic Cancer Research
KoGES: Korean Genome and Epidemiology Study
LD: linkage disequilibrium
MET: metabolic equivalent task
MR: Mendelian Randomization
MR‐PRESSO: Mendelian Randomisation Pleiotropy RESidual Sum and Outlier
NCC: National Cancer Centre
OR: odds ratio
ORC: obesity‐related cancer
SD: standard deviation
SMD: standardized mean difference
SNP: single nucleotide polymorphism
T2DM: type 2 diabetes mellitus
tsHR: time‐split hazard ratio
WC: waist circumference

## 1 Introduction

The prevalence of Type 2 diabetes mellitus (T2DM) in China has surged dramatically in the past decades, increasing from 22.56 million in 2000 to 140.87 million in 2021, with projections reaching 174.43 million by 2045 [1]. This is a substantial public health problem, with a high economic burden associated with treatment [2].

Among these concerns, there is evidence from many observational studies in European ancestry participants that T2DM is associated with increased risk of cancer, mainly among obesity‐related cancers (ORCs) [3]. However, it is unclear whether these associations are causal given multiple sources of bias [4]. Common biases include (i) immortal time bias when prevalent T2DM cases are used; (ii) detection time bias where much of the increased risk is identified in the early time window after T2DM diagnosis through various co‐detections of disease (e.g., new incidental diagnosis of diabetes in the clinic during work‐up for a cancer surgical resection); (iii) reverse causation, whereby undiagnosed cancer (particularly pancreatic or liver cancer) can induce diabetes as a paraneoplastic manifestation, creating the appearance that diabetes precedes cancer when in fact the cancer was already present; and (iv) confounding, for example, by key mutual risk factors like excess adiposity, commonly approximated by body mass index (BMI) [5, 6].

According to the International Agency for Research on Cancer (IARC), excess adiposity is an established risk factor for at least 13 ORCs. These include esophageal adenocarcinoma, cancers of the colorectum, liver, pancreas, kidney (renal cell), gallbladder, stomach cardia, thyroid, postmenopausal breast, endometrium, ovary, as well as meningioma and multiple myeloma [7]. The hypothesis for a specific association between T2DM and ORCs stems from shared underlying metabolic pathways. Both T2DM and obesity are characterized by insulin resistance, compensatory hyperinsulinemia, and alterations in the insulin‐like growth factor (IGF) axis, which may promote carcinogenesis through mitogenic and anti‐apoptotic effects [8]. Additionally, adipose tissue dysfunction in obesity leads to chronic low‐grade inflammation and altered secretion of adipokines (e.g., increased leptin, decreased adiponectin), creating a pro‐tumorigenic environment [9]. While T2DM shows associations with increased risk of many ORCs, the evidence that it causally mediates the carcinogenic effect of obesity is weak, raising questions about whether T2DM is an etiological contributor or merely a marker of shared underlying risk factors, particularly adiposity [10]. Additionally, the evidence synthesized from Mendelian Randomization (MR) studies do not support a causal interpretation of the observed associations of T2DM on most cancer risks, except for pancreatic, cervical, and endometrial cancers [10]. However, most genome‐wide association studies (GWAS) of T2DM susceptibility and cancer risk were conducted in European ancestry populations, such that it is difficult to generalize MR results across different populations with distinct genetic makeups or gene–environment interactions [11].

In the Chinese population, observational studies have also identified T2DM as a risk factor for several cancer types including liver, pancreatic, colorectal, endometrial, and postmenopausal breast cancers [12, 13, 14, 15]. The study designs were either diabetes cohorts standardized against the Chinese population [12, 13, 14] or cohort studies using prevalent T2DM at baseline [15]—these are susceptible to immortal time bias. Cohort studies also encounter problems of adjusting for BMI measured after T2DM diagnosis. This may not reflect the pre‐existing adiposity status of participants as weight changes frequently occur post T2DM diagnosis, influenced by metabolic alterations, intentional weight loss efforts, and treatments aimed at managing blood glucose levels [16]. Thus, pre‐T2DM BMI measurements might provide a more accurate representation of long‐term adiposity exposure.

In this study, within the China Kadoorie Biobank (CKB), we aimed to assess the causal relevance of new‐onset T2DM and cancer incidence (see Text S1 for the full rationale for studying a Chinese population). We designed a matched cohort study to reduce the above‐mentioned biases by: (i) employing a sequential longitudinal matching on age, study region, and most importantly, baseline BMI, to address confounding due to adiposity; (ii) including only new‐onset T2DM cases to minimize immortal time bias; (iii) restricting analysis in pre‐diagnosis BMI to mitigate reverse causation; and (iv) accounting for detection time bias. In addition, we utilized two‐sample MR to strengthen causal interpretations.

## 2 Materials and Methods

### 2.1 Conventional Observational Analysis

#### 2.1.1 Study Population

The details of the study design of CKB have been reported elsewhere (http://www.ckbiobank.org/). The baseline survey recruited 512,724 participants aged 30–79 years between July 2004 and July 2008 from 10 representative geographical regions in China. Detailed information on demographic characteristics and physical measures was collected by trained health staff at each site. Further details on this cohort can be found in the *Cohort Profile* publication [17].

#### 2.1.2 Exposure

All information on non‐fatal incident disease status was attained through linkage with established disease registries and the national health insurance system and coded using the 10th Revision of the International Classification of Disease (ICD‐10). The exposure of interest was new‐onset diabetes (ICD‐10: E11–E14), assuming any incident diabetes identified during follow‐up was T2DM. We excluded participants with prevalent diabetes at baseline, determined through two methods as described in prior studies [18]– either physician‐reported as such at baseline or cohort‐detected based on levels of random or fasting plasma glucose, detailed in Figure S1.

#### 2.1.3 Covariates

Sex assigned at birth was self‐reported by participants and recorded at study entry. Anthropometric measurements were taken by trained health workers, with BMI calculated as weight (kg)/height (m^2^). The Working Group on Obesity in China suggests BMI thresholds of 24 kg/m^2^ for defining overweight and 28 kg/m^2^ for general obesity, respectively. Similarly, the Chinese Diabetes Society establishes waist circumference (WC) thresholds at 90 cm for men and 85 cm for women to define central obesity in the Chinese population [19]. These criteria were used here.

We used key demographic and lifestyle factors recorded at baseline as covariates: physical activity (in metabolic equivalent task [MET] hours, continuous), WC (continuous), smoking and alcohol consumption (ever regular: YES/NO), education (≥ 6 years: YES/NO), and annual household income (≥ 35,000 yuan: YES/NO). We also adjusted for cancer‐specific risk factors—for example, for liver cancer, we adjusted for cirrhosis/chronic hepatitis history (YES/NO). The selection of these covariates was informed by existing background knowledge of potential confounders in this context.

#### 2.1.4 Outcome Measures

We derived first primary incident cancers using ICD‐10 codes C00 to C97. CKB reported only 16 specific cancer types, and we grouped the following cancer sites into ORCs—cancers of the colon and rectum, liver, pancreas in both sexes, and postmenopausal breast, endometrium, and ovary in women (full codes in Table S1)—given prior evidence linking these sites to excess adiposity and our study's focus on disentangling the effects of T2DM from those of obesity. Postmenopausal breast cancer was defined as a diagnosis in women over 50 years due to the lack of reported menopause status in CKB [20]. Since histological and anatomical details were not available, we did not differentiate between esophageal adenocarcinoma (EAD) and squamous cell carcinoma, nor cardia gastric cancer and non‐cardia gastric cancer. In China, > 85% of esophageal cancers are squamous‐cell and two‐thirds of gastric cancers are non‐cardia [21, 22], both of which have weak or inconsistent links to obesity; therefore cancers of the esophagus (ICD‐10: C15) and stomach (ICD‐10: C16) were excluded from ORCs.

Secondary outcomes were composites of cancers, as follows: digestive tract cancers, digestive accessory organ cancers, and ORCs in female reproductive organs (Table S1).

Participants were censored at the earliest of first primary cancer diagnosis, death, loss to follow‐up, or administrative end of follow‐up (31 December 2018); for matched unexposed individuals, censoring additionally occurred at incident T2DM diagnosis (as detailed below).

#### 2.1.5 Matching

To reduce immortal time bias, we used a sequential longitudinal matching design separately per sex [23]. A 1:3 matching without replacement was implemented to enhance statistical power while ensuring most exposed individuals would be able to find at least one match.

Matching on baseline BMI was a key objective. The sequential longitudinal matching repeated the matching process throughout the follow‐up period, starting from the date of first incident T2DM diagnosis and continuing enrolment whenever a new participant was clinically diagnosed with T2DM (i.e., the index date) (Figure S2A) [23]. All participants who remained on the study and had not yet been diagnosed with T2DM or any cancer at each index date were initially eligible for matching. The main effect of interest was analogous to the “per‐protocol” effect in a trial, with matched individuals in the unexposed group being followed up until the diagnosis of T2DM or first primary cancer (whichever came first) for consistency with our study question (Figure S2B).

The overall eligibility of a potential unexposed match was defined by the following criteria: remained in study, remained undiagnosed with T2DM or cancer, from the same study region, at a similar age on the index date with the exposed individual (±3 years), and had a similar baseline BMI with the exposed individual, defined by a caliper width of 0.25 times the standard deviation (SD).

#### 2.1.6 Statistical Analysis

In 1:3 matching, any T2DM case that found at least one suitable match was retained; therefore, the final analytic set included slightly fewer than three unexposed matches per case. To display covariate balance, we applied pair‐matching under the same algorithm and calculated the standardized mean difference (SMD) for each baseline variable; |SMD| < 0.10 denoted acceptable balance [24]. Sex‐ and region‐specific T2DM incidence rates were age‐standardized to the baseline age distribution of the entire CKB population (age bands: < 40, 40–49, 50–59, 60–69, ≥ 70 years) to enable fair comparison across regions.

Cancer risks associated with new‐onset T2DM were estimated using a Cox model, with time since diagnosis (or matched time for unexposed) as the time scale, stratified by the matched set. The model specifically estimated time‐split coefficients for the exposure by incorporating an interaction term between exposure and follow‐up time windows, which were divided into periods of within 12 months and beyond 12 months. This division was based on the distribution of Schoenfeld residuals plotted against follow‐up time to account for early detection time bias. The primary reported outputs were time‐split hazard ratios (denoted as tsHRs) and their 95% confidence intervals (CIs) for time windows exceeding 12 months. A univariable model (Model 1) was initially fitted, complemented by a multivariable model adjusting for WC, smoking status, alcohol consumption habit, physical activity, highest education level, annual household income, and cirrhosis/chronic hepatitis history (Model 2). All analyses were conducted separately for men and women.

To account for differing rates of non‐cancer deaths in exposed versus matched unexposed, we developed competing risks models with non‐cancer death and first cancers at sites other than the outcome of interest treated as competing events. Fine–Gray subdistribution hazard models, stratified on the matched set and adjusted as in Model 2, produced subdistribution time‐split hazard ratios (sub‐tsHRs) for the > 12 month window; these were compared with the cause‐specific tsHRs from the main Cox analysis [25].

Sensitivity analyses were performed as follows: (i) lower the BMI caliper to 0.10 × SD; (ii) restrict to women who never smoked and never consumed alcohol to minimize residual confounding; and (iii) repeat the analysis under a 1:5 matching ratio.

### 2.2 MR

Two‐sample MR utilizing summary‐level genetic data from publicly available sources was applied in the present study (Figure S3). Specifically, genetic instruments for T2DM were extracted from the largest trans‐ethnic meta‐analysis available, with effect estimates, allele frequencies, and standard errors derived from East Asian summary statistics [26]. For cancer outcomes, we obtained genetic association data from the following East Asian sources: breast and endometrial cancers from the Korean Genome and Epidemiology Study (KoGES; 424 and 355 cases, respectively) [27]; colorectal cancer from the BBJ‐CRC study (6,692 cases) [28]; pancreatic cancer from the combined Japanese study with contributions from the Japan Pancreatic Cancer Research (JaPAN), National Cancer Centre (NCC) and BBJ (2,039 cases) [29]; and additional data for colorectal, pancreatic, breast, endometrial, liver, lung, and stomach cancers from the CKB genotyped subset (912, 205, 655, 102, 770, 1,802, and 888 cases, respectively). Full references for each GWAS are provided in Table S2. Specifically, from CKB, we derived genetic association data, adjusted for age, sex, and 10 genetic principal components for these seven site‐specific cancers among 100,640 participants genotyped with the bespoke CKB Axiom array. The genotyped subset was assembled in two waves that deliberately over‐sampled major incident cases of stroke, myocardial infarction and chronic obstructive pulmonary disease (COPD) but also included large random sets of participants, so its age‐, sex‐ and region‐specific distributions remain broadly representative of the parent cohort after weighting [30]. Details in sources of genetic summaries for the traits used in the present study are presented in Table S2. Single nucleotide polymorphisms (SNPs) that met the genome‐wide statistical significance threshold (*p* < $5\times 10^{-8}$) and were retained after linkage disequilibrium (LD) clumping (*R* ^2^ = 0.001, distance = 10 Mb) were proposed as initial genetic instruments for T2DM (*N* = 193). These selected SNPs altogether explained approximately 38.9% variance associated with T2DM (pseudo‐*R* ^2^ on the continuous liability scale). The details for calculations in R^2^, F‐statistics, and statistical power are provided in Text S2. Detailed information for SNPs is presented in Table S3. The inverse‐variance weighted (IVW) method was used as the main analytic approach because, when all instruments are valid (or pleiotropy is balanced), it is the most efficient and well‐powered estimator of the causal effect [31]. To probe robustness to horizontal pleiotropy, we pre‐specified a suite of sensitivity estimators with complementary assumptions: the weighted median (consistent if ≥ 50% of the weight comes from valid instruments), the weighted mode (robust when the largest cluster of instruments identifies the true effect), and MR‐Egger (which can detect/adjust for directional pleiotropy under the InSIDE assumption, albeit with lower power) [32]. We additionally used Mendelian Randomization Pleiotropy RESidual Sum and Outlier (MR‐PRESSO) test to identify and correct for outlier variants indicative of horizontal pleiotropy [33]. Further, to minimize the influence of pleiotropy via BMI, for each of the 193 T2DM‐instrument SNPs, we extracted SNP–BMI associations from the Taiwan Biobank GWAS and performed multivariable MR using the IVW method to estimate the direct effect of T2DM on each cancer while conditioning on BMI [34]. To assess instrument strength in the multivariable MR analyses, we calculated Sanderson–Windmeijer conditional F‐statistics for T2DM and BMI. Conditional F‐statistics were used to evaluate the strength of the instrument set for each exposure after accounting for correlations between exposures, with values greater than 10 conventionally indicating adequate conditional instrument strength. Finally, to assess potential reverse causation, we additionally conducted a reverse two‐sample MR analysis treating pancreatic cancer as the exposure and T2DM as the outcome. Instrumental variables for pancreatic cancer were obtained from the same Japanese GWAS indicated above comprising 2039 pancreatic cancer cases and 32,592 controls [29]. Owing to the limited number of available instruments (*N* = 3), only the IVW estimator was applied. For interpretability, all odds ratios (ORs) and 95% CIs were rescaled to represent the change in cancer risk per doubling in the odds of T2DM [35].

All *p* values were two‐sided, with *p* < 0.05 considered as statistically significant; data analyses were carried out using R, version 4.2.1 (R Foundation) using the following packages: *tidyverse* (version 4.2.2), *survival* (version 3.4.0), *cmprsk* (version 2.2.12), *TwoSampleMR* (version 0.6.8), *MVMR* (version 0.4.1), *ieugwasr* (version 1.0.1), *metafor* (version 4.6.0) and *forestplot* (version 3.1.6).

### 2.3 Triangulation of Observational and MR Data by Cancer Sites

We triangulated the data from observational and MR studies with a traffic‐light framework. Concordant significant estimates received a green rating. Discordance, where one estimate was non‐significant with the CI crossing the null, received an amber rating. Finally, red indicated consistent non‐significance across both methods.

## 3 Results

### 3.1 Baseline Characteristics Pre‐Matching

The derivation of 480,138 participants eligible for matching is shown in Figure S4. The baseline characteristics by men and women prior to matching as shown in Table S4. Compared with individuals with no diabetes at follow‐up, individuals with new‐onset T2DM were older, had lower education levels, higher family wealth, higher rates of obesity, and less physically active at baseline. The age‐standardized incidence rate of T2DM showed large variations across the ten study regions in both sexes, for example, the incidence rate in Zhejiang was more than three times higher than that in Gansu (Figure S5). The distributions of age at T2DM diagnosis and baseline BMI for exposed participants are shown in Figure S6 and Figure S7, respectively.

### 3.2 Key Characteristics Post‐Matching

After 1:3 matching, 8657 men and 13,680 women with new‐onset T2DM were included, matched to 25,852 and 40,938 unexposed individuals, respectively. The distributions of index age, region and baseline BMI values were well‐balanced (Table 1). There were 37 men and 36 women in the exposed group who failed to find any match. These “failed” cases were outliers in terms of diagnostic age for T2DM and(or) baseline BMI (Table S5).

**TABLE 1:** Key characteristics after paired exact matching.

Characteristic	Men			Women		
	New‐onset T2DM	Matched unexposed	SMD^c^	New‐onset T2DM	Matched unexposed	SMD^c^
	(*N* = 8,675)	(*N* = 8,675)		(*N* = 13,689)	(*N* = 13,689)	
Follow‐up time, median (IQR), months	48.9 (24.8–81.1)	50.0 (26.0–81.5)		53.2 (27.7–86.7)	53.0 (27.6–85.4)	
Index age, mean (SD), years	62.0 (10.1)	61.9 (10.1)		62.3 (9.6)	62.2 (9.5)	
Body mass index, mean (SD), kg/m^2^	25.5 (3.4)	25.5 (3.3)	0.010	25.8 (3.6)	25.7 (3.6)	0.012
Study region, *N* (%)						
Qingdao	622 (7.2)	622 (7.2)		957 (7.0)	957 (7.0)	
Harbin	1186 (13.7)	1186 (13.7)		1650 (12.1)	1650 (12.1)	
Haikou	473 (5.5)	473 (5.5)		746 (5.4)	746 (5.4)	
Suzhou	1040 (12.0)	1040 (12.0)		1634 (11.9)	1634 (11.9)	
Liuzhou	894 (10.3)	894 (10.3)		1127 (8.2)	1127 (8.2)	
Sichuan	912 (10.5)	912 (10.5)		1828 (13.4)	1828 (13.4)	
Gansu	376 (4.3)	376 (4.3)		543 (4.0)	543 (4.0)	
Henan	501 (5.8)	501 (5.8)		993 (7.3)	993 (7.3)	
Zhejiang	1645 (19.0)	1645 (19.0)		2634 (19.2)	2634 (19.2)	
Hunan	1026 (11.8)	1026 (11.8)		1577 (11.5)	1577 (11.5)	
Other baseline covariates						
Waist circumference, mean (SD), cm	88.0 (9.9)	87.0 (9.9)	0.096	84.4 (9.7)	83.5 (9.7)	0.097
≥ 6 years of education, *N* (%)	4887 (56.3)	4867 (56.1)	0.005	4530 (33.1)	4649 (34.0)	−0.018
Annual household income ≥ 35,000 yuan, *N* (%)	2272 (26.2)	2294 (26.4)	−0.006	2591 (18.9)	2601 (19.0)	−0.002
Ever regular smokers^a^, *N* (%)	6477 (74.7)	6335 (73.0)	0.037	662 (4.8)	620 (4.5)	0.015
Ever regular alcohol drinkers^b^, *N* (%)	3364 (38.8)	3508 (40.4)	−0.034	367 (2.7)	419 (3.1)	−0.023
Physical activity, mean (SD), MET h/d	20.8 (15.0)	21.3 (14.9)	−0.035	19.3 (12.8)	19.7 (12.8)	−0.038
History of cirrhosis/chronic hepatitis, *N* (%)	172 (2.0)	135 (1.6)	0.032	131 (1.0)	115 (0.8)	0.012

*Note:* This table presents descriptive characteristics for the 1:1 matched set used only for covariate balance assessment. The main analyses were performed using the full 1:3 matching design.

Abbreviations: IQR, interquartile range; MET, metabolic equivalent task; SMD, standardised mean difference.

^a^ Ever regular smokers included former and current regular smokers.

^b^ Ever regular alcohol drinkers included former regular and current weekly drinkers.

^c^ An absolute value less than 0.10 denoted acceptable balance.

### 3.3 Associations Between New‐Onset T2DM and Cancer Risks

There was evidence for early detection time bias. Crude risk ratios (RRs) for total cancers in exposed individuals compared to the matched unexposed were higher in the first year but remained relatively stable afterwards in both men and women (Table 2). In addition, when fitting a Cox model with a constant coefficient for the exposure, the Schoenfeld residuals plotted against follow‐up time showed a similar pattern (Figure S8). Therefore, we put a cutoff point at 12 months, and the tsHRs corresponding to the time window after 12 months were presented.

**TABLE 2:** Unadjusted total cancer IRs in new‐onset T2DM and in the matched unexposed, and crude RRs by annual time windows since index date following 1:3 exact matching.

			Men		Women	
			New‐onset T2DM	Matched unexposed	New‐onset T2DM	Matched unexposed
			(*N* = 8,657)	(*N* = 25,852)	(*N* = 13,680)	(*N* = 40,938)
Since index date	0–1 year	No. of cases	147	195	139	205
		Crude IR (95% CI)^a^	275.3 (230.8–320.8)	125.7 (108.1–143.4)	195.0 (162.3–227.0)	95.0 (81.9–108.0)
		Crude RR (95% CI)	2.19 (1.77–2.71)	1 (ref)	2.05 (1.65–2.54)	1 (ref)
	1–2 years	No. of cases	79	151	90	200
		Crude IR (95% CI)^a^	161.9 (126.2–197.6)	106.3 (89.3–123.2)	140.8 (111.7–170.0)	100.0 (86.1–113.9)
		Crude RR (95% CI)	1.52 (1.16–2.00)	1 (ref)	1.41 (1.10–1.81)	1 (ref)
	2–3 years	No. of cases	61	124	86	197
		Crude IR (95% CI)^a^	99.5 (74.5–124.5)	64.9 (53.5–76.3)	90.4 (71.3–109.5)	68.2 (58.7–77.8)
		Crude RR (95% CI)	1.54 (1.13–2.08)	1 (ref)	1.33 (1.03–1.71)	1 (ref)
	3–4 years	No. of cases	67	131	66	148
		Crude IR (95% CI)^a^	141.4 (107.5–175.2)	89.5 (74.2–104.9)	90.4 (68.6–112.2)	66.1 (55.4–76.8)
		Crude RR (95% CI)	1.58 (1.18–2.12)	1 (ref)	1.35 (1.01–1.80)	1 (ref)
	4–5 years	No. of cases	43	123	53	138
		Crude IR (95% CI)^a^	94.1 (66.0–122.2)	85.5 (70.4–100.6)	71.2 (52.1–90.4)	61.5 (51.3–71.7)
		Crude RR (95% CI)	1.10 (0.78–1.56)	1 (ref)	1.17 (0.85–1.61)	1 (ref)

*Note:* Only the first 5 years of follow‐up are shown to illustrate early risk patterns; total incident cancer cases over the full follow‐up period are presented in Table 3.

Abbreviations: IR, incidence rate; RR, risk ratio.

^a^ Per 1000 person‐years.

During follow‐up to 31 December 2018, there were 542 total first primary cancers in men with T2DM versus 964 in unexposed matches, and 582 in women with T2DM versus 1262 in unexposed matches. T2DM was associated with increased risk of total cancers in men (tsHR 1.57, 95% CI 1.38–1.78) and women (tsHR 1.29, 95% CI 1.14–1.46). In addition, men with new‐onset T2DM exhibited elevated risks for ORCs (tsHR 1.73, 95% CI 1.37–2.19), digestive tract organ cancers (tsHR 1.33, 95% CI: 1.06–1.67), and for digestive accessory organ cancers (liver and pancreas) (tsHR 2.23, 95% CI 1.59–3.11), as detailed in Table 3. Women with new‐onset T2DM had higher risks for ORCs (tsHR 1.34, 95% CI 1.10–1.62), and for digestive accessory organ cancers (liver and pancreas) (tsHR 2.94, 95% CI 1.94–4.45). However, T2DM had no significant effect on the risk for ORCs in reproductive organs, including endometrial, ovarian, and postmenopausal breast cancers (Table 3).

**TABLE 3:** Associations of new‐onset T2DM with risk of composite cancer groups following 1:3 exact matching.

		Men		Women	
		New‐onset T2DM	Matched unexposed	New‐onset T2DM	Matched unexposed
No. of participants		8,657	25,852	13,680	40,938
No. of person‐years		39,705	120,475	67,303	200,507
Total first primary cancers					
No. of cases		542	964	582	1,262
IR^a^		1365.1 (1250.1–1480.0)	800.2 (749.7–850.7)	864.7 (794.5–935.0)	629.4 (594.7–664.1)
tsHR (95% CI)^b^	Model 1^c^	1.58 (1.39–1.79)	1 (ref)	1.32 (1.17–1.49)	1 (ref)
	Model 2^d^	1.57 (1.38–1.78)	1 (ref)	1.29 (1.14–1.46)	1 (ref)
ORCs^e^					
No. of cases		176	279	222	520
IR^a^		443.3 (377.8–508.8)	231.6 (204.4–258.8)	329.9 (286.5–373.2)	259.3 (237.1–281.6)
tsHR (95% CI)^b^	Model 1^c^	1.81 (1.44–2.28)	1 (ref)	1.36 (1.12–1.65)	1 (ref)
	Model 2^d^	1.73 (1.37–2.19)	1 (ref)	1.34 (1.10–1.62)	1 (ref)
Total cancers minus ORCs					
No. of cases		366	685	360	742
IR^a^		921.8 (827.4–1016.2)	568.6 (526.0–611.2)	534.9 (479.6–590.1)	370.1 (343.4–396.7)
tsHR (95% CI)^b^	Model 1^c^	1.49 (1.28–1.74)	1 (ref)	1.30 (1.12–1.50)	1 (ref)
	Model 2^d^	1.49 (1.28–1.74)	1 (ref)	1.27 (1.09–1.47)	1 (ref)
Digestive tract organ cancers^f^					
No. of cases		161	360	127	286
IR^a^		405.5 (342.9–468.1)	298.8 (267.9–329.7)	188.7 (155.9–221.5)	142.6 (126.1–159.2)
tsHR (95% CI)^b^	Model 1^c^	1.34 (1.07–1.67)	1 (ref)	1.18 (0.93–1.51)	1 (ref)
	Model 2^d^	1.33 (1.06–1.67)	1 (ref)	1.14 (0.89–1.46)	1 (ref)
Digestive accessory organ cancers^g^					
No. of cases		104	117	75	100
IR^a^		261.9 (211.6–312.3)	97.1 (79.5–114.7)	111.4 (86.2–136.7)	49.9 (40.1–59.6)
tsHR (95% CI)^b^	Model 1^c^	2.36 (1.71–3.25)	1 (ref)	2.84 (1.91–4.22)	1 (ref)
	Model 2^d^	2.23 (1.59–3.11)	1 (ref)	2.94 (1.94–4.45)	1 (ref)
ORCs in reproductive organs^h^					
No. of cases				79	262
IR^a^				117.4 (91.5–143.3)	130.7 (114.8–146.5)
tsHR (95% CI)^b^	Model 1^c^			0.92 (0.69–1.21)	1 (ref)
	Model 2^d^			0.90 (0.68–1.20)	1 (ref)

Abbreviations: IR, incidence rate; ORCs, obesity‐related cancers; tsHR, time‐split hazard ratio.

^a^ Per 100,000 person‐years.

^b^ The tsHR for follow‐up time window over 12 months was presented.

^c^ Model 1 was stratified on the matched set.

^d^ Model 2 was stratified on the matched set and further adjusted for waist circumference, smoking status, alcohol consumption habit, physical activity, highest education level, annual household income, and cirrhosis/chronic hepatitis history (for composite cancer risks that encompassed liver cancer).

^e^ ORCs include cancers of the colon and rectum, liver, pancreas, and (in women) postmenopausal breast, endometrium, and ovary.

^f^ Digestive tract organ cancers include cancers of the esophagus, stomach, colon, and rectum.

^g^ Digestive accessory organ cancers refer to combined liver and pancreatic cancers.

^h^ ORCs in reproductive organs (women only) include postmenopausal breast, endometrial, and ovarian cancers.

In both men and women, significantly positive associations between new‐onset T2DM and site‐specific cancer risks were observed in liver (men tsHR 2.12, 95% CI 1.42–3.15; women tsHR 2.39, 95% CI 1.42–4.04) and pancreas (men tsHR 2.57, 95% CI 1.35–4.92; women tsHR 3.95, 95% CI 1.92–8.13). Notably, an elevated risk in lung cancer was observed in men (tsHR 1.55, 95% CI 1.17–2.05) but not in women (tsHR 1.15, 95% CI 0.87–1.52) (Table 4). For the other cancer sites tested, there were no significant associations.

**TABLE 4:** Associations of new‐onset T2DM with risk of major site‐specific cancers following 1:3 exact matching.

		Men		Women	
		New‐onset T2DM	Matched unexposed	New‐onset T2DM	Matched unexposed
No. of participants		8,657	25,852	13,680	40,938
No. of person‐years		39,705	120,475	67,303	200,507
Liver					
No. of cases		68	86	43	68
IR^a^		171.3 (130.6–212.0)	71.4 (56.3–86.5)	63.9 (44.8–83.0)	33.9 (25.9–42.0)
tsHR (95% CI)^b^	Model 1^c^	2.29 (1.57–3.34)	1 (ref)	2.40 (1.46–3.93)	1 (ref)
	Model 2^d^	2.12 (1.42–3.15)	1 (ref)	2.39 (1.42–4.04)	1 (ref)
Pancreas					
No. of cases		36	32	33	33
IR^a^		90.7 (61.1–120.3)	26.6 (17.4–35.8)	49.0 (32.3–65.8)	16.5 (10.8–22.1)
tsHR (95% CI)^b^	Model 1^c^	2.54 (1.38–4.68)	1 (ref)	2.70 (1.47–4.95)	1 (ref)
	Model 2^d^	2.57 (1.35–4.92)	1 (ref)	3.95 (1.92–8.13)	1 (ref)
Colorectum					
No. of cases		62	143	64	145
IR^a^		156.2 (117.3–195.0)	118.7 (99.2–138.2)	95.1 (71.8–118.4)	72.3 (60.5–84.1)
tsHR (95% CI)^b^	Model 1^c^	1.39 (0.98–1.98)	1 (ref)	1.18 (0.84–1.67)	1 (ref)
	Model 2^d^	1.33 (0.93–1.91)	1 (ref)	1.16 (0.82–1.64)	1 (ref)
Lung					
No. of cases		116	202	101	212
IR^a^		292.2 (239.0–345.3)	167.7 (144.5–190.8)	150.1 (120.8–179.3)	105.7 (91.5–120.0)
tsHR (95% CI)^b^	Model 1^c^	1.52 (1.15–2.00)	1 (ref)	1.19 (0.90–1.57)	1 (ref)
	Model 2^d^	1.55 (1.17–2.05)	1 (ref)	1.15 (0.87–1.52)	1 (ref)
Stomach					
No. of cases		60	132	40	89
IR^a^		151.1 (112.9–189.4)	109.6 (90.9–128.3)	59.4 (41.0–77.9)	44.4 (35.2–53.6)
tsHR (95% CI)^b^	Model 1^c^	1.16 (0.79–1.70)	1 (ref)	1.16 (0.75–1.78)	1 (ref)
	Model 2^d^	1.13 (0.77–1.68)	1 (ref)	1.11 (0.72–1.71)	1 (ref)
Breast (overall)					
No. of cases				68	197
IR^a^				101.0 (77.0–125.0)	98.3 (84.5–112.0)
tsHR (95% CI)^b^	Model 1^c^			1.04 (0.77–1.41)	1 (ref)
	Model 2^d^			1.01 (0.73–1.38)	1 (ref)
Postmenopausal breast					
No. of cases				62	188
IR^a^				92.1 (69.2–115.1)	93.8 (80.4–107.2)
tsHR (95% CI)^b^	Model 1^c^			1.02 (0.74–1.40)	1 (ref)
	Model 2^d^			1.02 (0.74–1.41)	1 (ref)

Abbreviations: IR, incidence rate; tsHR, time‐split hazard ratio.

^a^ Per 100,000 person‐years.

^b^ The tsHR for follow‐up time window over 12 months was presented.

^c^ Model 1 was stratified on the matched set.

^d^ Model 2 was stratified on the matched set and further adjusted for waist circumference, smoking status, alcohol consumption habit, physical activity, highest education level, annual household income, and cirrhosis/chronic hepatitis history (for liver cancer).

### 3.4 The Competing Risk Model

The cumulative incidence of total first primary cancers and competing non‐cancer deaths before a potential cancer diagnosis (new‐onset T2DM vs. matched unexposed) was illustrated in Figure S9. We also examined the censoring patterns in the two groups following 1:3 exact matching (Table S6). While cumulative cancer incidence in the exposed group overrode that in the unexposed matches, competing risks from non‐cancer deaths showed a similar trend, indicating that the tsHRs derived from a cause‐specific hazard model could have overestimated the relative risk in cancer incidence in a real‐world setting. In support of this, competing risks modelling resulted in attenuated sub‐tsHRs compared to cause‐specific tsHRs from the main model by different degrees, but conclusions regarding effect directions remained unchanged (Table S7).

### 3.5 Sensitivity Analyses

We applied a narrower caliper for BMI matching (0.1 SD, 1:3 matching), excluding 84 men and 74 women without suitable matches. Results remained consistent with the main analyses where a caliper equal to 0.25 SD was applied (Table S8 and Table S9). Using 1:5 matching, more exposed individuals remained unmatched, but results were generally consistent with 1:3 matching. An exception was a significantly positive association observed in lung cancer for women (tsHR 1.33, 95% CI 1.02–1.73) (Table S10 and Table S11).

Among women eligible for matching at baseline, 61.2% were naïve to both cigarette and alcohol. In 9083 exposed women with no history of tobacco or alcohol use, 31 lacked matches. Results aligned with the main analysis, showing significant associations between new‐onset T2DM and ORCs (tsHR 1.39, 95% CI 1.10–1.76), and digestive accessory organ cancers (liver and pancreas) (tsHR 2.05, 95% CI 1.34–3.12) (Table S12). As shown in Table S13, for site‐specific cancers tested, a significantly positive signal was only observed for cancer in the liver (tsHR 1.83, 95% CI 1.08–3.12) (the data for pancreatic cancer were not shown due to limited case number).

### 3.6 MR

In univariable MR, among the site‐specific cancers tested, genetic liability to T2DM was associated with higher odds of pancreatic cancer at a nominally significant level (pooled OR 1.08, 95% CI 1.02–1.15, *p* = 0.01) (Figure S10). This association remained robust in sensitivity analyses (weighted median, weighted mode, MR‐Egger, and MR‐PRESSO; Table S14) and after multivariable adjustment for BMI (OR 1.07, 95% CI 1.01–1.14; Figure 1). In multivariable MR, the conditional F‐statistics for T2DM ranged from 79.1 to 81.2 across analyses, indicating strong conditional instrument strength (Table S15). For all other cancer sites examined—colorectum, breast, endometrium, liver, lung, and stomach—the confidence intervals crossed the null and *p* values exceeded 0.05 (Figure S10 and Figure 1). However, the estimation precision is low for some cancer types, and the absence of significance might be due to the lack of statistical power (Table S16). In reverse MR using pancreatic cancer liability as the exposure and T2DM as the outcome, no statistically significant association was observed (IVW OR 1.06, 95% CI 0.98–1.14, *p* = 0.18; Figure S11). However, only three independent pancreatic cancer susceptibility variants were available as instrumental variables, limiting statistical power and precluding robust sensitivity analyses; therefore, a modest reverse causal effect cannot be excluded.

**FIGURE 1:**
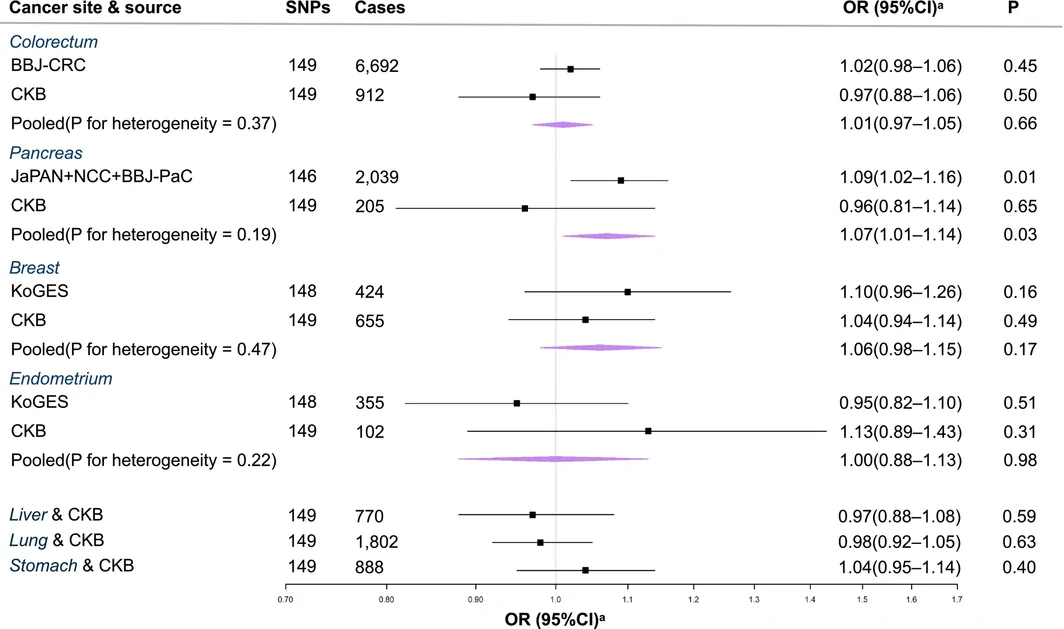
Multivariable MR with adjustments for BMI of the associations of T2DM with major site‐specific cancers in East Asians. Abbreviations: T2DM, type 2 diabetes mellitus; BBJ‐CRC, Biobank Japan colorectal cancer cohort; CKB, China Kadoorie Biobank; JaPAN, Japan pancreatic cancer research; NCC, national cancer centre; BBJ‐PaC, Biobank Japan pancreatic cancer cohort; KoGES, Korean genome and epidemiology study; OR, odds ratio; SNPs, single nucleotide polymorphisms. ^a^Estimates indicating OR of cancer per doubling in odds of T2DM.

### 3.7 Triangulation of Evidence

Concordant significance (green) was achieved solely for pancreatic cancer. An amber rating was assigned to colorectal, breast, and stomach cancers, where neither analytical approach provided a statistically significant estimate. Discordance (red) was identified for two cancer sites: lung cancer, where a sex‐specific (in men) association in the observational analysis was absent in the MR results, and liver cancer, where a positive observational finding lacked support from MR (Table 5).

**TABLE 5:** Triangulation of evidence from matched‐cohort analysis in CKB and multivariable MR analysis in East Asians for the casual effect of T2DM on site‐specific cancer risks.

Cancer site	Effect estimates			Interpretation	Bias diagnoses
	tsHR^a^		OR^b^		
	Men	Women			
Pancreas	2.57 (1.35–4.92)	3.95 (1.92–8.13)	1.07 (1.01–1.14)	Concordant significance	Residual confounding from alcohol related exposures could result in inflated effect magnitude in tsHR.
Colorectum	1.33 (0.93–1.91)	1.16 (0.82–1.64)	1.01 (0.97–1.05)	Concordant non‐significance	Residual confounding from alcohol related exposures could result in inflated effect magnitude in tsHR.
Breast		1.01 (0.73–1.38)^c^	1.06 (0.98–1.15)	Concordant non‐significance	The limited number of cancer cases in both analyses increases the imprecision of the effect estimates. This is reflected in the wide 95% CIs that cross the null value of 1.
Stomach	1.13 (0.77–1.68)	1.11 (0.72–1.71)	1.04 (0.95–1.14)	Concordant non‐significance	The limited number of cancer cases in both analyses increases the imprecision of the effect estimates. This is reflected in the wide 95% CIs that cross the null value of 1.
Liver	2.12 (1.42–3.15)	2.39 (1.42–4.04)	0.97 (0.88–1.08)	Discordant	Restricted power in MR could lead to the failure to detect a small effect; residual confounding from major contributors to incident liver cancer in China (e.g., HBV/HCV infections) could result in inflated effect magnitude in tsHR.
Lung	1.55 (1.17–2.05)	1.15 (0.87–1.52)	0.98 (0.92–1.05)	Discordant	The only notable inconsistency was a meaningful tsHR estimate above 1 observed in men. This result could be biased by smoking‐related confounding. Future investigations using sex‐specific MR and/or factorial MR to test for exposure–smoking interactions—when statistical power allows—could provide new insights into this issue.

*Note:* The significance threshold is *P* < 0.05. Statistical significance was defined as two‐sided *P* < 0.05. Green: Concordant significance — both the observational analysis and MR provided statistically significant associations (*P* < 0.05) in the same direction. Amber: Concordant non‐significance — neither the observational analysis nor MR provided a statistically significant association (both estimates crossed the null with *P* ≥ 0.05). Red: Discordant — the observational analysis showed a statistically significant association (*P* < 0.05), but the MR estimate was null (crossed the null and was not statistically significant, *P* ≥ 0.05).

Abbreviations: HBV, hepatitis B virus; HCV, hepatitis C virus; OR, odds ratio; tsHR, time‐split hazard ratio.

^a^ Observational estimate from the multivariable‐adjusted Cox model following 1:3 matching, indicating the tsHR in cancer in new‐onset T2DM vs. matched unexposed.

^b^ Multivariable MR estimate indicating the OR in cancer per doubling in odds of T2DM after adjusting for BMI.

^c^ tsHR for overall breast cancer in women.

## 4 Discussion

### 4.1 Key Findings

Our analyses suggest that, after minimizing key biases, new‐onset T2DM is associated with a higher risk of total cancers and, in particular, cancers of the liver and pancreas in Chinese adults. MR further supported a causal effect of genetic liability to T2DM on pancreatic cancer, whereas evidence for other sites was weak. Triangulating the observational and genetic results therefore supports a credible causal interpretation for pancreatic cancer but not for other cancers.

### 4.2 Comparison With Previous Studies and Potential Mechanisms

Earlier analyses in the CKB that relied on prevalent T2DM reported higher risks of total, liver, pancreatic, and breast cancer [15]. In contrast, by prospectively identifying incident T2DM, matching chronologically to reduce immortal time bias, using pre‐diagnosis BMI as a proxy for long‐term adiposity, and accounting for early detection time bias via time‐split coefficients, we again observed positive associations for liver and pancreatic cancers but no association for postmenopausal breast cancer. In cancers affecting both sexes, we observe positive associations for lung and digestive‐tract cancers in men but largely null estimates in women—likely reflecting residual confounding by smoking and alcohol, exposures that are far more common among men compared with women in China [17]. Results in women who were naïve to tobacco and alcohol were consistent with the main analyses, reinforcing this interpretation and motivating larger analyses among smoking‐ and alcohol‐naïve men.

No association was observed for postmenopausal breast cancer, aligning with western patterns reported in UK Biobank or Sister Study [36, 37]. Limited cases precluded reliable estimates for endometrial and ovarian cancer, but the composite outcome (postmenopausal breast, endometrium, ovary) also showed null association. These hormone‐related cancers appear to be driven more by adiposity than by downstream metabolic dysfunctions such as T2DM [38]. Consistent with prior CKB findings that suggest positive associations between multiple adiposity indicators and these cancer types, our results—a null association after matching on baseline BMI to account for adiposity‐related confounding—suggest that obesity, rather than T2DM itself, may be the primary driver of elevated risk for these three cancers [15, 39].

For liver cancer, chronic hepatitis B/C virus (HBV/HCV) infection remains a dominant etiology in China [40]. Our adjustment for a history of cirrhosis/chronic hepatitis at baseline provided limited information on active HBV/HCV infection, so residual confounding by hepatitis status remains possible. Furthermore, the MR analysis in this context was underpowered to detect small effects (only 770 liver cancer cases from CKB). Both of these sources of bias could lead to the discordant conclusion indicated by evidence triangulation (Table 5). Therefore, considering the strong signal in the observational estimate, we believe it is premature to reject an association between T2DM and liver cancer. We argue for targeted future research to reduce major confounding and increase the statistical power of MR analyses. For gastric cancer, our observational estimates could plausibly be biased upward by unmeasured *Helicobacter pylori* , which may be more prevalent in individuals with T2DM [41]; limited site‐specific case numbers further constrained precision. For colorectal cancer, our observational estimates—after accounting for early detection bias—do not indicate a robust association with new‐onset T2DM, consistent with attenuation reported when early follow‐up is excluded in a prior study [15]. Overall, the pattern emerging across digestive sites is that signals are strongest and most coherent for pancreas, weaker for liver (and potentially confounded by hepatitis), and largely null elsewhere after addressing early detection bias and confounding by adiposity and lifestyle.

The MR results sharpen this picture. Across site‐specific cancers with available summary statistics in East Asians, genetically proxied T2DM was associated with pancreatic cancer risk and showed little evidence for associations with colorectum, breast, endometrium, liver, lung, or stomach. The consistency between MR and our bias‐minimized cohort analysis for pancreas supports a causal role of T2DM in pancreatic carcinogenesis. For other sites, discordance (e.g., observationally positive but MR‐null for liver) suggests residual confounding in observational estimates or limited MR power, given modest case numbers for some cancers (e.g., liver cancer).

### 4.3 Strengths and Limitations

This study has several strengths. First, we focused exclusively on incident T2DM with covariates recorded at baseline, preserving correct temporality. Our sex‐specific, sequential longitudinal matching on age, region, and baseline BMI directly targeted confounding by adiposity and improved exchangeability, while region matching helped mitigate geographic variation in unmeasured factors (dietary patterns, environmental exposures, and healthcare access). By explicitly modelling an early post‐diagnosis window, we demonstrated the inflation of effect estimates within 12 months of T2DM onset—consistent with reverse causality and detection bias—and reported effects beyond that window. Second, we also quantified subdistribution hazard ratios to reflect effect magnitudes in the presence of competing non‐cancer deaths, which is more informative for clinical prediction and planning. Third, we did extensive sensitivity analyses and showed no significant deviation from our results under different model assumptions. Finally, triangulating with MR allowed us to separate likely causal from non‐causal associations.

Limitations should be acknowledged. First, statistical power in matched‐cohort analysis was limited for several sites due to the strict exposure definition and follow‐up beginning at the index date, and some sites (e.g., thyroid, gallbladder) were absent or recorded without histological detail (e.g., esophagus and stomach), preventing analyses by subtype. Second, we lacked repeated measures of time‐varying covariates prior to diabetes onset; reliance on a single baseline BMI may introduce measurement error and obscure adiposity trajectories that better capture lifetime exposure. Third, relatively short follow‐up length constrained our ability to model risks across multiple post‐diagnosis durations [42]; prior evidence suggests duration of T2DM may modify liver and pancreatic cancer risks in Chinese populations, so extended observation in CKB will be informative [43, 44]. Fourth, residual confounding cannot be fully excluded—especially for smoking and alcohol in men—as implied by sex differences. Fifth, the statistical power in MR analyses for some cancers was modest, particularly for endometrial cancer and breast cancer, where we had limited power to detect small‐to‐modest effects (Table S16). Therefore, the absence of a significant genetic association does not definitively rule out small effects for these sites. Larger, population‐based cancer GWAS in East Asians shall enable more precise estimates in future studies. Finally, while we addressed correlated pleiotropy through multivariable MR adjusting for BMI—the most important shared risk factor—residual confounding by unmeasured heritable factors cannot be excluded. Emerging methods such as CAUSE, MR‐Horse, and LHC‐MR offer promising approaches to distinguish causal effects from correlated pleiotropy without requiring measured confounders; these may be valuable in future studies when fuller genome‐wide summary statistics become available for East Asian populations [45, 46, 47].

### 4.4 Clinical Implications

The broader implications are twofold. First, the convergence of observational and genetic evidence for pancreas argues that T2DM is part of the causal pathway for pancreatic cancer in East Asians. This strengthens the case for heightened vigilance and risk‐adapted strategies among high‐risk T2DM subgroups—particularly those with additional risk factors (e.g., older age at diabetes onset, obesity, heavy smoking or alcohol use, and chronic liver disease)—rather than blanket cancer screening for all people with T2DM. Second, for most other cancers, excess risks observed in people with T2DM likely reflect shared upstream determinants (notably adiposity and lifestyle) rather than diabetes per se, underscoring the primacy of prevention targeting weight, diet, and smoking/alcohol, alongside equitable access to routine, guideline‐recommended screening in the general population.

### 4.5 Future Research Directions

Future research should refine estimates of cumulative pre‐diabetes adiposity—for example, incorporating BMI/WC trajectories from childhood and early adulthood—to reduce exposure misclassification and improve matched designs [48, 49]. Larger studies are needed to test effect modification by age at T2DM onset and BMI category, which were infeasible under caliper matching here but are clinically relevant for targeting interventions. Given the genetic and phenotypic heterogeneity of T2DM, integrating partitioned polygenic risk scores with phenotype‐guided subtyping could reveal T2DM clusters with distinct cancer risks and mechanisms, improving risk stratification and informing mechanistically tailored prevention [50, 51].

## 5 Conclusion

In conclusion, after addressing key sources of bias and triangulating with MR, we find robust evidence consistent with a causal association between T2DM and pancreatic cancer in Chinese adults, with weaker and likely non‐causal associations for most other sites. These results argue for targeted, risk‐adapted cancer prevention within diabetes care and renewed emphasis on upstream determinants—particularly adiposity and smoking/alcohol—rather than treating T2DM as a broadly carcinogenic exposure.

## Author Contributions

**Mengying Wang:** methodology, formal analysis, writing – original draft, conceptualization. **Owen Tipping:** methodology, writing – review and editing, conceptualization. **Bowen Liu:** methodology, writing – review and editing, conceptualization. **Derrick A. Bennett:** methodology, writing – review and editing, conceptualization. **Baiyong Shen:** methodology, writing – review and editing, conceptualization. **Siyi Zou:** methodology, writing – review and editing, conceptualization. **Shulin Zhao:** methodology, writing – review and editing, conceptualization. **Richard M. Martin:** methodology, supervision, writing – review and editing, conceptualization. **Matthew Sperrin:** methodology, supervision, writing – review and editing, conceptualization. **Andrew G. Renehan:** conceptualization, methodology, funding acquisition, writing – review and editing, supervision, project administration.

## Funding

This study has been delivered through the NIHR Manchester Biomedical Research Centre (BRC) (NIHR203308). M.S. is supported by the UK Research and Innovation (UKRI) and the Engineering and Physical Sciences Research Council (EPSRC) for CHAI—EPSRC AI Hub for Causality in Healthcare AI with Real Data [grant number EP/Y028856/1]. R.M.M. is a National Institute for Health Research Senior Investigator (NIHR202411). R.M.M. is supported by a Cancer Research UK (C18281/A29019) programme grant (the Integrative Cancer Epidemiology Programme). R.M.M. is also supported by the NIHR Bristol Biomedical Research Centre which is funded by the NIHR (BRC‐1215‐20011) and is a partnership between University Hospitals Bristol and Weston NHS Foundation Trust and the University of Bristol.

## Ethics Statement

This study is a secondary analysis of anonymized data from CKB. The original CKB study obtained ethical approval from the Ethical Review Committee of the Chinese Centre for Disease Control and Prevention (Beijing, China; Ethics ID: 005/2004) and the Oxford Tropical Research Ethics Committee, University of Oxford (UK; Ethics ID: OxTREC 025–04). All CKB participants gave written informed consent. The MR analyses used publicly available GWAS summary statistics; no further ethical approval was required.

## Conflicts of Interest

The authors declare no conflicts of interest.

## Supporting information


**Figure S1:** Flowchart showing the confirmation of prevalent diabetes cases.
**Figure S2:** Illustration of the longitudinal matching design in the present study.
**Figure S3:** Illustration of the study design in Mendelian randomization.
**Figure S4:** Flowchart for identifying eligible individuals for matching at baseline.
**Figure S5:** Age‐standardized incidence rate of T2DM in men and women across the 10 study regions.
**Figure S6:** Distribution of age at T2DM diagnosis among participants eligible for matching as exposed individuals.
**Figure S7:** Distribution of baseline body mass index among participants eligible for matching as exposed individuals.
**Figure S8:** Schoenfeld residuals for T2DM status from a standard Cox model with constant coefficients.
**Figure S9:** Cumulative incidence function for total first primary cancers and competing non‐cancer deaths.
**Figure S10:** Univariable Mendelian Randomization for the associations of T2DM with major site‐specific cancers in East Asians.
**Figure S11:** Forest plot of SNP‐specific MR estimates for the association between genetically predicted pancreatic cancer and risk of T2DM.
**Table S1:** Categorized cancer outcomes in the present study.
**Table S2:** Sources of genetic summaries for the traits used in the Mendelian Randomization part of the present study.
**Table S3:** Genetic instruments for T2DM used in the present study.
**Table S4:** Baseline characteristics of participants categorized by T2DM status during follow‐upa.
**Table S5:** Characteristics in participants with new‐onset T2DM who failed to find any match in 1:3.
**Table S6:** Examination in censoring patterns following 1:3 exact matching.
**Table S7:** Comparison of time‐split effect estimates from cause‐specific and subdistribution hazard models for T2DM on first primary cancer incidence.
**Table S8:** Associations of new‐onset T2DM with composite cancer risks after applying a narrower caliper width in BMI matching.
**Table S9:** Associations of new‐onset T2DM with site‐specific cancer risks after applying a narrower caliper width in BMI matching.
**Table S10:** Associations of new‐onset T2DM with composite cancer risks following 1:5 exact matching.
**Table S11:** Associations of new‐onset T2DM with site‐specific cancer risks following 1:5 exact matching.
**Table S12:** Associations of new‐onset T2DM with composite cancer risks in smoking‐ and alcohol‐naïve women.
**Table S13:** Associations of new‐onset T2DM with site‐specific cancer risks in smoking‐ and alcohol‐naïve women.
**Table S14:** MR sensitivity analyses of the associations between T2DM and major site‐specific cancers in East Asians.
**Table S15:** Sanderson–Windmeijer conditional F‐statistics for exposures included in multivariable Mendelian Randomization analyses.
**Table S16:** Power calculation for two‐sample Mendelian Randomisation of the associations between T2DM and site‐specific cancers.
**Text S1:** The rationale for studying T2DM–cancer casual associations in a Chinese population.
**Text S2:** Estimation of R^2^, F‐statistics, and statistical power for summary‐level MR.

## Data Availability

The China Kadoorie Biobank data resource analyzed in this study can be accessed by bona fide researchers here (https://www.ckbiobank.org/data‐access). Ancestry‐specific GWAS summary statistics for T2DM can be obtained from the DIAGRAM consortium (https://www.diagram‐consortium.org/downloads.html). BBJ‐CRC colorectal cancer sub‐study, BMI data and the combined Japanese study for pancreatic cancer data are publicly available under GWAS with accession GCST005591, GCST90103751, and via the JaPAN consortium (http://www.aichi‐med‐u.ac.jp/JaPAN/current_initiatives‐e.html). Further data that support the findings of this study are available from the corresponding author upon request.
