# Supplementary material for: New‐Onset Type 2 Diabetes Mellitus and Cancer Risk: A Matched Cohort Study in China Kadoorie Biobank
Source: Int J Cancer. 2026 Jul 9;159(11):2705–19. doi: 10.1002/ijc.70638 (PMC13637062; doi:10.1002/ijc.70638)
Supplement: Supplementary file 1 [file IJC-159-2705-s001.docx]

**New-onset type 2 diabetes mellitus and cancer risk: a matched cohort study in China Kadoorie Biobank**

Authors

Mengying Wang, Owen Tipping, Bowen Liu, Derrick A. Bennett, Baiyong Shen, Siyi Zou, Shulin Zhao, Richard M. Martin, Matthew Sperrin, Andrew G. Renehan

**Table of Contents**

[Text S1: The rationale for studying T2DM–cancer casual associations in a Chinese population	3](#_Toc233998807)

[Text S2: Estimation of R^2^, F-statistics, and statistical power for summary-level MR	5](#_Toc233998808)

[Table S1: Categorised cancer outcomes in the present study	6](#_Toc233998809)

[Table S2: Sources of genetic summaries for the traits used in the Mendelian Randomisation part of the present study	7](#_Toc233998810)

[Table S3: Genetic instruments for T2DM used in the present study	8](#_Toc233998811)

[Table S4: Baseline characteristics of participants categorised by T2DM status during follow-up^a^	14](#_Toc233998812)

[Table S5: Characteristics in participants with new-onset T2DM who failed to find any match in 1:3 matching	15](#_Toc233998813)

[Table S6: Examination in censoring patterns following 1:3 exact matching	16](#_Toc233998814)

[Table S7: Comparison of time-split effect estimates from cause-specific and subdistribution hazard models for T2DM on first primary cancer incidence	17](#_Toc233998815)

[Table S8: Associations of new-onset T2DM with composite cancer risks after applying a narrower caliper width in BMI matching	18](#_Toc233998816)

[Table S10: Associations of new-onset T2DM with composite cancer risks following 1:5 exact matching	20](#_Toc233998817)

[Table S11: Associations of new-onset T2DM with site-specific cancer risks following 1:5 exact matching	21](#_Toc233998818)

[Table S12: Associations of new-onset T2DM with composite cancer risks in smoking- and alcohol-naïve women	22](#_Toc233998819)

[Table S13: Associations of new-onset T2DM with site-specific cancer risks in smoking- and alcohol-naïve women	23](#_Toc233998820)

[Table S14: MR sensitivity analyses of the associations between T2DM and major site-specific cancers in East Asians	24](#_Toc233998821)

[Table S15: Sanderson–Windmeijer conditional F-statistics for exposures included in multivariable Mendelian Randomisation analyses	26](#_Toc233998822)

[Table S16: Power calculation for two-sample Mendelian Randomisation of the associations between T2DM and site-specific cancers^a^	27](#_Toc233998823)

[Figure S1: Flowchart showing the confirmation of prevalent diabetes cases	28](#_Toc233998824)

[Figure S2: Illustration of the longitudinal matching design in the present study	29](#_Toc233998825)

[Figure S3: Illustration of the study design in Mendelian Randomisation	30](#_Toc233998826)

[Figure S4: Flowchart for identifying eligible individuals for matching at baseline	31](#_Toc233998827)

[Figure S5: Age-standardised incidence rate of T2DM in men and women across the ten study regions	32](#_Toc233998828)

[Figure S6: Distribution of age at T2DM diagnosis among participants eligible for matching as exposed individuals	33](#_Toc233998829)

[Figure S7: Distribution of baseline body mass index among participants eligible for matching as exposed individuals	34](#_Toc233998830)

[Figure S8: Schoenfeld residuals for T2DM status from a standard Cox model with constant coefficients	35](#_Toc233998831)

[Figure S9: Cumulative incidence function for total first primary cancers and competing non-cancer deaths	36](#_Toc233998832)

[Figure S10: Univariable Mendelian Randomisation for the associations of T2DM with major site-specific cancers in East Asians	37](#_Toc233998833)

[Figure S11: Forest plot of SNP-specific MR estimates for the association between genetically predicted pancreatic cancer and risk of T2DM	38](#_Toc233998834)

[References	39](#_Toc233998835)

## Text S1: The rationale for studying T2DM–cancer casual associations in a Chinese population

Why the Chinese population is unique for studying T2DM–cancer associations?

1. Distinct disease burden and epidemiological transition: China is undergoing a rapid epidemiological transition, facing a “double burden” of disease with rising non-communicable diseases (NCDs) while still contending with infectious disease sequelae. The prevalence of T2DM in China has increased dramatically—from 22.56 million in 2000 to 140.87 million in 2021, with projections reaching 174.43 million by 2045. Simultaneously, cancer patterns in China differ from Western populations, with higher rates of liver, stomach, and oesophageal cancer, and lower rates of certain hormone-related cancers.^1^ This unique disease landscape provides an opportunity to examine associations that may be masked or diluted in Western populations.
2. Recent multi-ancestry studies have demonstrated that the genetic architecture of T2DM differs substantially between East Asian and European populations. For example, East Asians exhibit stronger effect sizes for certain T2DM risk loci (such as KCNQ1) and a higher proportion of lipodystrophy-related polygenic risk, whereas Europeans show a more polygenic pattern with smaller individual effects. Additionally, the BMI threshold for equivalent T2DM risk is considerably lower in East Asians than in Europeans.^2^ These differences mean that findings from European-ancestry studies cannot be directly extrapolated to Chinese populations.
3. Distinct environmental and lifestyle exposures: The Chinese population exhibits unique patterns of key risk factors, e.g., low background rates of smoking and alcohol in women, different adiposity distribution and regional heterogeneity.
4. Limited existing evidence base: As noted in the Introduction, most existing evidence on T2DM and cancer comes from Western populations, and there is substantial uncertainty about its relevance to populations with different disease rates, risk exposures, and genetic susceptibilities.

**Would the biases be different in Chinese populations?**

The core sources of bias—immortal time bias, detection time bias, reverse causation, and confounding—are universal methodologic challenges that apply regardless of population. However, the magnitude and direction of these biases may differ in important ways:

1. Confounding by adiposity: Given the lower BMI thresholds at which metabolic risk manifests in East Asians, confounding by adiposity may be more pronounced and require tighter control.
2. Detection time bias: Healthcare access and screening practices vary considerably across different geographic regions in China. Additionally, the higher background incidence of certain cancers (e.g., liver cancer related to HBV/HCV) means that detection bias might differentially affect specific sites.
3. Reverse causation: For cancers that can induce diabetes as a paraneoplastic manifestation (particularly pancreatic cancer), the magnitude of reverse causation could differ if cancer stage at diagnosis differs between populations.
4. Residual confounding by lifestyle factors: The extreme sex differences in smoking and alcohol consumption in China create both challenges and opportunities. While residual confounding may affect estimates in men, the availability of a large smoking- and alcohol-naive female subgroup in CKB allows us to assess the robustness of findings in the absence of these confounders.

## Text S2: Estimation of R^2^, F-statistics, and statistical power for summary-level MR

To evaluate the strength of genetic instruments for T2DM, we estimated the proportion of variance in the exposure explained by each SNP ($R^{2}$) and the corresponding *F-statistics*.

$R^{2}$ was calculated based on effect alle frequency ($\mathrm{EAF}$) and the SNP-exposure effect size ($\beta$) using the following equation:

$$R^{2}=2 \cdot EAF \cdot\left( 1-EAF \right)\cdot\beta^{2}$$

*F-statistics* were calculated for each SNP using the following equation:

$$F=\frac{{\left( N_{eff}-1 \right)\cdot R}^{2}}{1-R^{2}}$$

where $N_{eff}$ is the effective sample size. A value of *F* >10 was considered indicative of a strong instrument. Details in the calculated $R^{2}$ and *F-statistics* per SNP were presented in **Table S3.**

To summarise instrument strength across all SNPs, a total *F-statistics* was calculated as:

$$F_{total}=\frac{\begin{aligned} \left( {N^{*}}_{eff}-K-1 \right)\cdot R_{total}^{2} \end{aligned}}{\begin{aligned} K\cdot(1-R_{total}^{2}) \end{aligned}}$$

where $K$ is the number of instruments, $R_{total}^{2}$ is the sum of individual $R^{2}$ values, and ${N^{*}}_{eff}$ is the harmonic mean of per-SNP effective sample sizes calculated as:

$$N_{eff}^{*}=\frac{n}{\sum_{i=1}^{n} \begin{aligned} \frac{1}{{\begin{aligned} (N_{eff}) \end{aligned}}_{i}} \end{aligned}}$$

$R_{total}^{2}$was then used in MR power calculations using an online calculator (<https://sb452.shinyapps.io/power/>) ^3^.

## Table S1: Categorised cancer outcomes in the present study

| **Cancer site** | **ICD-10 codes** | **Digestive tract organs** | **Digestive accessory organs** | **ORCs** | | **ORCs in reproductive organs (women only)** |
| --- | --- | --- | --- | --- | --- | --- |
|  |  |  |  | **Men** | **Women** |  |
| All malignant neoplasms | C00–C97 |  |  |  |  |  |
| Lip, oral cavity and pharynx | C00–C14 | ● |  |  |  |  |
| Oesophagus | C15 | ● |  |  |  |  |
| Stomach | C16 | ● |  |  |  |  |
| Colon and rectum | C18–C20 | ● |  | ● | ● |  |
| Liver | C22 |  | ● | ● | ● |  |
| Pancreas | C25 |  | ● | ● | ● |  |
| Bronchus and lung | C33–C34 |  |  |  |  |  |
| Premenopausal breast | C50 |  |  |  |  |  |
| Postmenopausal breast | C50 |  |  |  | ● | ● |
| Cervix | C53 |  |  |  |  |  |
| Endometrium | C54.1 |  |  |  | ● | ● |
| Ovary | C56 |  |  |  | ● | ● |
| Prostate | C61 |  |  |  |  |  |
| Kidney | C64 |  |  | ● | ● |  |
| Bladder | C67 |  |  |  |  |  |
| Lymphoma | C81–C85 |  |  |  |  |  |
| Leukaemia | C91–C95 |  |  |  |  |  |

Abbreviations: M, men; W, women; ORCs, obesity-related cancers; ICD-10, the International Classification of Disease, 10^th^ Revision.

## Table S2: Sources of genetic summaries for the traits used in the Mendelian Randomisation part of the present study

| **Trait** | **Cohort/Consortium** | **No. of participants** | **No. of cases** | **No. of controls** | **Reference** | **PMID** | **Sample source** |
| --- | --- | --- | --- | --- | --- | --- | --- |
| T2DM | DIAGRAM Consortium | 427,504 | 88,109 | 339,395 | Suzuki K, et al. (2024)^4^ | 38374256 | 40 GWAS in East Asians |
| BMI | TWB | 21,930 | NA | NA | Wong H, et al. (2022)^5^ | 35051171 | 21,930 Taiwanese from TWB |
| Colorectal cancer | BBJ-CRC study | 33,870 | 6,692 | 27,178 | Tanikawa C, et al. (2018)^6^ | 29471430 | BBJ-CRC Study (cases from BBJ, controls from three population-based cohorts) |
|  | CKB | 100,640 | 912 | 99,728 | NA | NA | 100,640 CKB participants with genotyping data at baseline |
| Pancreatic cancer | JaPAN+NCC+BBJ-PaC | 34,631 | 2,039 | 32,592 | Lin Y, et al. (2020)^7^ | 32581250 | Three Japanese studies: the JaPAN consortium, NCC, and the BBJ subcohort on pancreatic cancer (controls from four population-based cohorts) |
|  | CKB | 100,640 | 205 | 100,435 | NA | NA | 100,640 CKB participants with genotyping data at baseline |
| Breast cancer | KoGES | 46,330 | 424 | 45,906 | Nam K, et al (2022)^8^ | 36777999 | Three population-based prospective sub-cohorts in Korea |
|  | CKB | 57,625 | 655 | 56,970 | NA | NA | 57,652 female CKB participants with genotyping data at baseline |
| Endometrial cancer | KoGES | 46,330 | 355 | 45,975 | Nam K, et al (2022)^8^ | 36777999 | Three population-based prospective sub-cohorts in Korea |
|  | CKB | 57,625 | 102 | 57,523 | NA | NA | 57,652 female CKB participants with genotyping data at baseline |
| Liver cancer | CKB | 100,640 | 770 | 99,870 | NA | NA | 100,640 CKB participants with genotyping data at baseline |
| Lung cancer | CKB | 100,640 | 1,802 | 98,838 | NA | NA | 100,640 CKB participants with genotyping data at baseline |
| Stomach cancer | CKB | 100,640 | 888 | 99,752 | NA | NA | 100,640 CKB participants with genotyping data at baseline |

Abbreviations: NA, not applicable; T2DM, type 2 diabetes mellitus; GWAS, genome-wide association study; BMI, body mass index; TWB, Taiwan Biobank; BBJ-CRC, Biobank Japan colorectal cancer cohort; CKB, China Kadoorie Biobank; JaPAN, Japan pancreatic cancer research; NCC, national cancer centre; BBJ-PaC, Biobank Japan pancreatic cancer cohort; KoGES, Korean genome and epidemiology study.

## Table S3: Genetic instruments for T2DM used in the present study

| **SNP** | **CHR** | **POS^a^** | **Effect allele** | **Other allele** | **BETA** | **SE** | **EAF** | **P** | **No. of cases** | **No. of controls** | **N_eff_** | **R^2^** | **F-statistic** |
| --- | --- | --- | --- | --- | --- | --- | --- | --- | --- | --- | --- | --- | --- |
| rs75372910 | 1 | 20688361 | T | C | -0.0451 | 0.0066 | 0.368 | 9.69E-12 | 88109 | 339395 | 247159 | 0.0009 | 234 |
| rs12119086 | 1 | 24371508 | A | G | -0.0677 | 0.0124 | 0.157 | 4.96E-08 | 42726 | 207363 | 112063 | 0.0012 | 300 |
| rs755249 | 1 | 39995074 | T | C | 0.0599 | 0.0088 | 0.1591 | 9.31E-12 | 88109 | 339395 | 247159 | 0.0010 | 238 |
| rs10749857 | 1 | 46248143 | T | C | -0.0602 | 0.0099 | 0.2955 | 1.00E-09 | 42529 | 203027 | 111309 | 0.0015 | 374 |
| rs12354253 | 1 | 51148711 | A | G | -0.0977 | 0.0112 | 0.0926 | 3.12E-18 | 87912 | 335059 | 246405 | 0.0016 | 397 |
| rs2269242 | 1 | 64108682 | A | G | -0.0583 | 0.0082 | 0.1901 | 9.83E-13 | 87912 | 335059 | 246405 | 0.0010 | 259 |
| rs11588753 | 1 | 146714427 | C | G | 0.051 | 0.0078 | 0.2163 | 5.18E-11 | 88109 | 339395 | 247159 | 0.0009 | 218 |
| rs670323 | 1 | 177868990 | A | G | 0.0577 | 0.0077 | 0.217 | 8.79E-14 | 88109 | 339395 | 247159 | 0.0011 | 280 |
| rs10797937 | 1 | 184017131 | A | G | -0.0404 | 0.0064 | 0.5403 | 2.43E-10 | 88109 | 339395 | 247159 | 0.0008 | 201 |
| rs6689629 | 1 | 204539291 | A | G | 0.0399 | 0.0066 | 0.6064 | 1.48E-09 | 87912 | 335059 | 246405 | 0.0008 | 188 |
| rs340874 | 1 | 214159256 | T | C | -0.0485 | 0.0065 | 0.616 | 8.84E-14 | 88109 | 339395 | 247159 | 0.0011 | 275 |
| rs348330 | 1 | 229672955 | A | G | -0.0555 | 0.0073 | 0.3119 | 4.15E-14 | 87912 | 335059 | 246405 | 0.0013 | 327 |
| rs7564711 | 2 | 639061 | T | G | -0.1022 | 0.011 | 0.0941 | 1.41E-20 | 88109 | 339395 | 247159 | 0.0018 | 441 |
| rs28680411 | 2 | 16237427 | A | G | 0.0425 | 0.0072 | 0.3984 | 3.88E-09 | 87950 | 337771 | 246579 | 0.0009 | 214 |
| rs1260326 | 2 | 27730940 | T | C | -0.0708 | 0.0064 | 0.5415 | 9.03E-29 | 88109 | 339395 | 247159 | 0.0025 | 617 |
| rs12712928 | 2 | 45192080 | C | G | 0.0545 | 0.0066 | 0.4017 | 1.83E-16 | 88109 | 339395 | 247159 | 0.0014 | 353 |
| rs243018 | 2 | 60586707 | C | G | -0.0578 | 0.007 | 0.3338 | 9.60E-17 | 88109 | 339395 | 247159 | 0.0015 | 368 |
| rs10184881 | 2 | 65684152 | A | G | 0.0515 | 0.0091 | 0.8493 | 1.39E-08 | 88109 | 339395 | 247159 | 0.0007 | 168 |
| rs3731600 | 2 | 120231070 | C | G | 0.1282 | 0.0191 | 0.9568 | 2.12E-11 | 83923 | 315971 | 234546 | 0.0014 | 336 |
| rs2167500 | 2 | 121370591 | A | C | 0.0374 | 0.0067 | 0.4901 | 1.93E-08 | 88109 | 339395 | 247159 | 0.0007 | 173 |
| rs4499362 | 2 | 149568396 | T | C | -0.043 | 0.0067 | 0.3611 | 1.85E-10 | 88109 | 339395 | 247159 | 0.0009 | 211 |
| rs75536691 | 2 | 165381518 | A | G | 0.1875 | 0.0214 | 0.9753 | 2.08E-18 | 85983 | 337970 | 244518 | 0.0017 | 419 |
| rs75179644 | 2 | 213687103 | T | C | 0.0746 | 0.011 | 0.8673 | 1.13E-11 | 88109 | 339395 | 247159 | 0.0013 | 317 |
| rs117809958 | 2 | 234191103 | A | T | 0.2703 | 0.0251 | 0.023 | 4.02E-27 | 87950 | 337771 | 246579 | 0.0033 | 814 |
| rs2067819 | 3 | 12359049 | A | G | -0.1069 | 0.0166 | 0.045 | 1.17E-10 | 85087 | 312200 | 236279 | 0.0010 | 243 |
| rs3860593 | 3 | 23102368 | A | G | -0.0587 | 0.0085 | 0.826 | 3.84E-12 | 88109 | 339395 | 247159 | 0.0010 | 245 |
| rs56979234 | 3 | 23436567 | A | G | -0.1115 | 0.0084 | 0.1801 | 4.09E-40 | 88109 | 339395 | 247159 | 0.0037 | 911 |
| rs704361 | 3 | 63883671 | T | G | -0.0829 | 0.0067 | 0.3514 | 1.48E-35 | 88109 | 339395 | 247159 | 0.0031 | 777 |
| rs12490891 | 3 | 84783138 | A | G | 0.0366 | 0.0065 | 0.3892 | 2.26E-08 | 88109 | 339395 | 247159 | 0.0006 | 158 |
| rs9860393 | 3 | 114966421 | A | G | 0.0446 | 0.0069 | 0.6891 | 8.07E-11 | 88109 | 339395 | 247159 | 0.0009 | 211 |
| rs6767514 | 3 | 123151762 | A | C | -0.053 | 0.0068 | 0.3474 | 7.09E-15 | 88109 | 339395 | 247159 | 0.0013 | 315 |
| rs13073553 | 3 | 125091755 | A | G | -0.0358 | 0.0065 | 0.5041 | 4.24E-08 | 87912 | 335059 | 246405 | 0.0006 | 158 |
| rs9873341 | 3 | 152387723 | T | C | 0.0474 | 0.0071 | 0.2782 | 2.05E-11 | 88109 | 339395 | 247159 | 0.0009 | 223 |
| rs9875793 | 3 | 170686573 | A | G | 0.061 | 0.0082 | 0.8151 | 1.31E-13 | 88109 | 339395 | 247159 | 0.0011 | 278 |
| rs4389513 | 3 | 185506857 | T | G | -0.129 | 0.0068 | 0.6882 | 2.95E-79 | 88109 | 339395 | 247159 | 0.0071 | 1778 |
| rs6444187 | 3 | 186674138 | C | G | -0.0421 | 0.0065 | 0.5252 | 6.93E-11 | 88109 | 339395 | 247159 | 0.0009 | 219 |
| rs34307738 | 3 | 195818265 | A | G | -0.0442 | 0.0066 | 0.3849 | 2.88E-11 | 88109 | 339395 | 247159 | 0.0009 | 229 |
| rs730831 | 4 | 1240299 | T | G | 0.099 | 0.0069 | 0.6806 | 3.93E-46 | 87912 | 335059 | 246405 | 0.0043 | 1058 |
| rs4865473 | 4 | 1782586 | A | G | -0.0694 | 0.0077 | 0.7062 | 1.51E-19 | 84327 | 328752 | 238303 | 0.0020 | 495 |
| rs4234731 | 4 | 6299914 | A | G | -0.1106 | 0.018 | 0.0613 | 7.77E-10 | 85754 | 336542 | 242392 | 0.0014 | 348 |
| rs10938398 | 4 | 45186139 | A | G | 0.0399 | 0.007 | 0.294 | 1.13E-08 | 88109 | 339395 | 247159 | 0.0007 | 163 |
| rs12499651 | 4 | 71854281 | A | G | 0.0713 | 0.0079 | 0.2065 | 2.26E-19 | 88109 | 339395 | 247159 | 0.0017 | 412 |
| rs117624659 | 4 | 85339618 | T | C | 0.2015 | 0.0209 | 0.9699 | 5.16E-22 | 87950 | 337771 | 246579 | 0.0024 | 587 |
| rs6813195 | 4 | 153520475 | T | C | -0.0773 | 0.0064 | 0.4907 | 1.35E-33 | 88109 | 339395 | 247159 | 0.0030 | 740 |
| rs6885132 | 5 | 14768092 | C | G | 0.0409 | 0.0065 | 0.555 | 2.86E-10 | 88109 | 339395 | 247159 | 0.0008 | 204 |
| rs878608 | 5 | 36246700 | T | C | -0.0563 | 0.0086 | 0.8282 | 6.62E-11 | 88109 | 339395 | 247159 | 0.0009 | 223 |
| rs72756138 | 5 | 50085981 | T | C | 0.056 | 0.0095 | 0.1276 | 4.15E-09 | 88109 | 339395 | 247159 | 0.0007 | 173 |
| rs3762991 | 5 | 52085403 | T | C | 0.0437 | 0.0069 | 0.3094 | 1.78E-10 | 88109 | 339395 | 247159 | 0.0008 | 202 |
| rs256904 | 5 | 55810305 | A | T | -0.0706 | 0.0064 | 0.5075 | 1.11E-28 | 88109 | 339395 | 247159 | 0.0025 | 617 |
| rs6453133 | 5 | 74692776 | A | G | 0.0413 | 0.0064 | 0.4881 | 9.69E-11 | 88109 | 339395 | 247159 | 0.0009 | 211 |
| rs6453436 | 5 | 78626464 | A | G | -0.0358 | 0.0064 | 0.4248 | 2.65E-08 | 88109 | 339395 | 247159 | 0.0006 | 155 |
| rs7721099 | 5 | 87936379 | T | C | -0.0385 | 0.0064 | 0.5378 | 2.10E-09 | 88109 | 339395 | 247159 | 0.0007 | 182 |
| rs3853212 | 5 | 95849348 | T | C | -0.0406 | 0.0064 | 0.5811 | 2.94E-10 | 88109 | 339395 | 247159 | 0.0008 | 199 |
| rs154120 | 5 | 111375061 | A | G | 0.0352 | 0.0064 | 0.5536 | 4.05E-08 | 88109 | 339395 | 247159 | 0.0006 | 151 |
| rs12657328 | 5 | 122651711 | T | C | 0.0632 | 0.0116 | 0.9073 | 4.73E-08 | 88109 | 339395 | 247159 | 0.0007 | 166 |
| rs451643 | 5 | 176511432 | T | G | 0.0531 | 0.0065 | 0.434 | 4.15E-16 | 85780 | 337056 | 242491 | 0.0014 | 343 |
| rs9379084 | 6 | 7231843 | A | G | -0.076 | 0.0084 | 0.2221 | 1.57E-19 | 88109 | 339395 | 247159 | 0.0020 | 494 |
| rs9350271 | 6 | 20683164 | A | G | 0.1947 | 0.0064 | 0.4218 | 1.06E-202 | 88109 | 339395 | 247159 | 0.0185 | 4656 |
| rs879882 | 6 | 31139452 | T | C | -0.0605 | 0.0064 | 0.5666 | 2.91E-21 | 88109 | 339395 | 247159 | 0.0018 | 445 |
| rs9265745 | 6 | 31302718 | A | G | 0.043 | 0.0064 | 0.4845 | 1.86E-11 | 87912 | 335059 | 246405 | 0.0009 | 228 |
| rs35800511 | 6 | 32636433 | A | G | 0.0485 | 0.008 | 0.7694 | 1.26E-09 | 83710 | 327595 | 236922 | 0.0008 | 206 |
| rs118012224 | 6 | 34205822 | T | C | 0.1156 | 0.0106 | 0.8927 | 9.75E-28 | 88109 | 339395 | 247159 | 0.0026 | 634 |
| rs742762 | 6 | 39046644 | A | C | 0.0801 | 0.0072 | 0.7132 | 1.15E-28 | 88109 | 339395 | 247159 | 0.0026 | 650 |
| rs62405419 | 6 | 50787459 | T | G | 0.0515 | 0.0072 | 0.2695 | 8.41E-13 | 88109 | 339395 | 247159 | 0.0010 | 258 |
| rs80196932 | 6 | 117996631 | T | C | 0.056 | 0.0079 | 0.7798 | 1.49E-12 | 88109 | 339395 | 247159 | 0.0011 | 266 |
| rs4273712 | 6 | 126964510 | A | G | -0.0568 | 0.0064 | 0.5319 | 5.64E-19 | 87117 | 337721 | 244667 | 0.0016 | 398 |
| rs2297638 | 6 | 131940890 | A | C | -0.0477 | 0.0067 | 0.6555 | 9.82E-13 | 88109 | 339395 | 247159 | 0.0010 | 254 |
| rs12662968 | 6 | 137288760 | A | T | 0.0558 | 0.0065 | 0.4608 | 1.09E-17 | 88109 | 339395 | 247159 | 0.0015 | 383 |
| rs7768451 | 6 | 157003689 | A | C | -0.0562 | 0.0085 | 0.8308 | 3.90E-11 | 88109 | 339395 | 247159 | 0.0009 | 220 |
| rs4709741 | 6 | 164092291 | A | C | -0.0665 | 0.011 | 0.1053 | 1.69E-09 | 88109 | 339395 | 247159 | 0.0008 | 206 |
| rs12523813 | 6 | 166223997 | T | C | -0.07 | 0.0127 | 0.9216 | 3.29E-08 | 87912 | 335059 | 246405 | 0.0007 | 175 |
| rs17168486 | 7 | 14898282 | T | C | 0.0627 | 0.0065 | 0.4194 | 4.57E-22 | 87912 | 335059 | 246405 | 0.0019 | 474 |
| rs10244051 | 7 | 15063833 | T | G | -0.067 | 0.0068 | 0.3193 | 7.33E-23 | 88109 | 339395 | 247159 | 0.0020 | 483 |
| rs3735567 | 7 | 28219310 | A | G | -0.0638 | 0.0078 | 0.2243 | 1.87E-16 | 88001 | 338685 | 246784 | 0.0014 | 351 |
| rs2240404 | 7 | 30707302 | A | G | -0.0461 | 0.0083 | 0.1946 | 2.70E-08 | 88109 | 339395 | 247159 | 0.0007 | 165 |
| rs35452727 | 7 | 44266184 | T | C | 0.0555 | 0.0085 | 0.1839 | 7.84E-11 | 87950 | 337771 | 246579 | 0.0009 | 229 |
| rs7790934 | 7 | 69701965 | A | G | -0.0649 | 0.0067 | 0.6633 | 3.82E-22 | 88109 | 339395 | 247159 | 0.0019 | 466 |
| rs39204 | 7 | 89734318 | A | C | 0.0679 | 0.0077 | 0.2244 | 7.78E-19 | 88109 | 339395 | 247159 | 0.0016 | 397 |
| rs2074120 | 7 | 93107093 | A | C | 0.0427 | 0.0068 | 0.3237 | 3.49E-10 | 88109 | 339395 | 247159 | 0.0008 | 197 |
| rs75990271 | 7 | 102336979 | T | C | 0.0693 | 0.0087 | 0.8034 | 1.68E-15 | 86692 | 331491 | 243167 | 0.0015 | 376 |
| rs2233580 | 7 | 127253550 | T | C | 0.3296 | 0.0114 | 0.0885 | 8.57E-183 | 86882 | 331561 | 243602 | 0.0175 | 4409 |
| rs3824004 | 7 | 127253551 | T | G | 0.2406 | 0.0174 | 0.036 | 1.62E-43 | 87912 | 335059 | 246405 | 0.0040 | 997 |
| rs62487901 | 7 | 140436560 | A | G | 0.0745 | 0.0122 | 0.082 | 1.19E-09 | 87164 | 330076 | 243803 | 0.0008 | 207 |
| rs887609 | 7 | 156794983 | A | G | 0.0647 | 0.0082 | 0.2203 | 4.26E-15 | 88109 | 339395 | 247159 | 0.0014 | 356 |
| rs6459732 | 7 | 156921020 | T | C | -0.0627 | 0.0108 | 0.1071 | 5.80E-09 | 88109 | 339395 | 247159 | 0.0008 | 186 |
| rs28412 | 8 | 17907387 | A | G | 0.074 | 0.013 | 0.066 | 1.31E-08 | 88109 | 339395 | 247159 | 0.0007 | 167 |
| rs56825414 | 8 | 36758946 | T | C | 0.0462 | 0.0076 | 0.2436 | 9.68E-10 | 88109 | 339395 | 247159 | 0.0008 | 195 |
| rs12680217 | 8 | 37397803 | T | C | 0.0462 | 0.0065 | 0.5546 | 1.03E-12 | 88109 | 339395 | 247159 | 0.0011 | 261 |
| rs6989203 | 8 | 41523745 | A | G | -0.1035 | 0.0093 | 0.1364 | 9.33E-29 | 88109 | 339395 | 247159 | 0.0025 | 625 |
| rs185063984 | 8 | 75203220 | A | G | -0.1337 | 0.0197 | 0.9697 | 1.18E-11 | 87955 | 338283 | 246619 | 0.0011 | 260 |
| rs12679044 | 8 | 95891356 | A | C | -0.0426 | 0.007 | 0.7127 | 1.12E-09 | 88109 | 339395 | 247159 | 0.0007 | 184 |
| rs13266634 | 8 | 118184783 | T | C | -0.1258 | 0.0064 | 0.4131 | 5.51E-85 | 88109 | 339395 | 247159 | 0.0077 | 1911 |
| rs2175055 | 9 | 1033354 | T | C | 0.0396 | 0.0065 | 0.4277 | 9.89E-10 | 88109 | 339395 | 247159 | 0.0008 | 190 |
| rs4237150 | 9 | 4290085 | C | G | 0.0846 | 0.0064 | 0.4289 | 1.22E-39 | 87912 | 335059 | 246405 | 0.0035 | 870 |
| rs10965250 | 9 | 22133284 | A | G | -0.1881 | 0.0064 | 0.4351 | 6.18E-189 | 88109 | 339395 | 247159 | 0.0174 | 4375 |
| rs7030811 | 9 | 22288140 | A | T | -0.1223 | 0.0144 | 0.0577 | 2.15E-17 | 87912 | 335059 | 246405 | 0.0016 | 403 |
| rs34532817 | 9 | 81914318 | T | C | -0.0931 | 0.0141 | 0.0556 | 4.29E-11 | 88109 | 339395 | 247159 | 0.0009 | 225 |
| rs10125947 | 9 | 83974536 | A | G | 0.0521 | 0.0067 | 0.6301 | 9.57E-15 | 88109 | 339395 | 247159 | 0.0013 | 313 |
| rs2796441 | 9 | 84308948 | A | G | -0.0792 | 0.0065 | 0.6085 | 1.01E-33 | 88109 | 339395 | 247159 | 0.0030 | 741 |
| rs113154802 | 9 | 98278413 | T | C | -0.069 | 0.0103 | 0.1154 | 1.73E-11 | 88109 | 339395 | 247159 | 0.0010 | 240 |
| rs28378473 | 9 | 139245460 | T | C | -0.1452 | 0.0119 | 0.1091 | 3.16E-34 | 84524 | 333088 | 239057 | 0.0041 | 1017 |
| rs11257655 | 10 | 12307894 | T | C | 0.1257 | 0.0065 | 0.4786 | 1.96E-84 | 88109 | 339395 | 247159 | 0.0079 | 1965 |
| rs57219676 | 10 | 12399515 | A | G | 0.0706 | 0.0119 | 0.0842 | 3.54E-09 | 88109 | 339395 | 247159 | 0.0008 | 190 |
| rs2815657 | 10 | 12548710 | A | G | 0.0423 | 0.0069 | 0.3348 | 6.92E-10 | 88109 | 339395 | 247159 | 0.0008 | 197 |
| rs117414485 | 10 | 23578177 | T | C | 0.1088 | 0.0151 | 0.047 | 5.86E-13 | 88109 | 339395 | 247159 | 0.0011 | 262 |
| rs77757273 | 10 | 63716319 | A | G | -0.0629 | 0.0095 | 0.1336 | 2.90E-11 | 88109 | 339395 | 247159 | 0.0009 | 227 |
| rs59050225 | 10 | 64950110 | T | C | -0.065 | 0.0078 | 0.2111 | 1.18E-16 | 88109 | 339395 | 247159 | 0.0014 | 348 |
| rs10824316 | 10 | 77295843 | T | C | -0.0432 | 0.0064 | 0.5164 | 1.01E-11 | 88109 | 339395 | 247159 | 0.0009 | 231 |
| rs703977 | 10 | 80944230 | T | G | 0.0614 | 0.0065 | 0.566 | 2.69E-21 | 85780 | 337056 | 242491 | 0.0019 | 459 |
| rs10887774 | 10 | 89766043 | A | G | 0.0412 | 0.0064 | 0.4636 | 9.36E-11 | 88109 | 339395 | 247159 | 0.0008 | 209 |
| rs11598977 | 10 | 94146580 | A | T | -0.1295 | 0.0148 | 0.9387 | 1.95E-18 | 88109 | 339395 | 247159 | 0.0019 | 478 |
| rs10882100 | 10 | 94460687 | C | G | 0.1418 | 0.0073 | 0.25 | 4.78E-84 | 88109 | 339395 | 247159 | 0.0075 | 1878 |
| rs10882890 | 10 | 99048262 | A | G | -0.0499 | 0.007 | 0.7083 | 1.33E-12 | 88109 | 339395 | 247159 | 0.0010 | 255 |
| rs13306146 | 10 | 112840171 | A | G | 0.0462 | 0.007 | 0.6949 | 5.06E-11 | 88109 | 339395 | 247159 | 0.0009 | 224 |
| rs7901695 | 10 | 114754088 | T | C | -0.2911 | 0.0162 | 0.9591 | 5.27E-72 | 87117 | 337721 | 244667 | 0.0066 | 1654 |
| rs11199805 | 10 | 122919206 | T | C | -0.0605 | 0.007 | 0.2941 | 7.42E-18 | 88109 | 339395 | 247159 | 0.0015 | 376 |
| rs11200595 | 10 | 124142629 | T | C | 0.0427 | 0.0066 | 0.3969 | 8.25E-11 | 88109 | 339395 | 247159 | 0.0009 | 216 |
| rs7482891 | 11 | 2197112 | A | G | 0.1107 | 0.0119 | 0.0809 | 1.60E-20 | 85516 | 334762 | 241548 | 0.0018 | 451 |
| rs7121454 | 11 | 2624456 | T | G | 0.0998 | 0.0107 | 0.8905 | 1.60E-20 | 88109 | 339395 | 247159 | 0.0019 | 481 |
| rs74728949 | 11 | 2758546 | T | C | -0.0603 | 0.0089 | 0.1592 | 1.57E-11 | 85780 | 337056 | 242491 | 0.0010 | 241 |
| rs2237897 | 11 | 2858546 | T | C | -0.2562 | 0.007 | 0.3654 | 7.13E-295 | 85780 | 337056 | 242491 | 0.0304 | 7760 |
| rs10832835 | 11 | 2880009 | A | C | 0.0576 | 0.0083 | 0.1964 | 5.01E-12 | 85583 | 332720 | 241737 | 0.0010 | 259 |
| rs2074314 | 11 | 17411821 | T | C | -0.0786 | 0.0065 | 0.6244 | 2.48E-33 | 88109 | 339395 | 247159 | 0.0029 | 718 |
| rs11030104 | 11 | 27684517 | A | G | 0.042 | 0.0064 | 0.5592 | 5.11E-11 | 88109 | 339395 | 247159 | 0.0009 | 215 |
| rs4562816 | 11 | 51247632 | A | T | 0.0387 | 0.0068 | 0.4876 | 1.01E-08 | 85193 | 330127 | 240411 | 0.0007 | 185 |
| rs628993 | 11 | 61539691 | A | G | 0.0382 | 0.0069 | 0.5065 | 2.81E-08 | 87737 | 336602 | 245867 | 0.0007 | 180 |
| rs602652 | 11 | 69462642 | A | G | 0.0509 | 0.0085 | 0.7978 | 1.83E-09 | 85780 | 337056 | 242491 | 0.0008 | 207 |
| rs11603349 | 11 | 72460694 | T | C | 0.1461 | 0.0143 | 0.9385 | 1.64E-24 | 88109 | 339395 | 247159 | 0.0025 | 610 |
| rs4331050 | 11 | 92696014 | T | G | 0.0561 | 0.009 | 0.4357 | 4.51E-10 | 42726 | 207363 | 112063 | 0.0015 | 383 |
| rs10750397 | 11 | 128234144 | A | G | 0.0401 | 0.0072 | 0.2653 | 3.13E-08 | 88109 | 339395 | 247159 | 0.0006 | 155 |
| rs4140660 | 12 | 4310220 | T | C | 0.0489 | 0.008 | 0.1998 | 1.11E-09 | 88109 | 339395 | 247159 | 0.0008 | 189 |
| rs3812821 | 12 | 4382324 | C | G | -0.0532 | 0.0071 | 0.5142 | 6.29E-14 | 87005 | 328452 | 243224 | 0.0014 | 350 |
| rs12578595 | 12 | 27964996 | T | C | -0.0679 | 0.0068 | 0.3338 | 1.43E-23 | 88109 | 339395 | 247159 | 0.0021 | 508 |
| rs80234489 | 12 | 31441179 | A | C | -0.1227 | 0.0085 | 0.8256 | 3.17E-47 | 88109 | 339395 | 247159 | 0.0043 | 1076 |
| rs7132908 | 12 | 50263148 | A | G | 0.043 | 0.0073 | 0.2573 | 3.97E-09 | 88109 | 339395 | 247159 | 0.0007 | 175 |
| rs2298640 | 12 | 50304574 | A | G | -0.0381 | 0.007 | 0.3181 | 4.80E-08 | 88109 | 339395 | 247159 | 0.0006 | 156 |
| rs2612059 | 12 | 66239111 | A | C | -0.0636 | 0.0067 | 0.6599 | 1.87E-21 | 88109 | 339395 | 247159 | 0.0018 | 450 |
| rs1913195 | 12 | 71416780 | T | G | -0.0445 | 0.0072 | 0.2771 | 6.46E-10 | 88109 | 339395 | 247159 | 0.0008 | 196 |
| rs10860209 | 12 | 97850215 | A | C | -0.0405 | 0.0065 | 0.4112 | 4.60E-10 | 88109 | 339395 | 247159 | 0.0008 | 196 |
| rs1426371 | 12 | 108629780 | A | G | -0.0485 | 0.0065 | 0.5116 | 7.26E-14 | 88109 | 339395 | 247159 | 0.0012 | 291 |
| rs2074356 | 12 | 112645401 | A | G | -0.0551 | 0.0094 | 0.1841 | 3.86E-09 | 87124 | 333814 | 244475 | 0.0009 | 226 |
| rs7132351 | 12 | 118414926 | A | G | 0.0734 | 0.0076 | 0.2435 | 2.95E-22 | 88109 | 339395 | 247159 | 0.0020 | 492 |
| rs118074491 | 12 | 121363506 | A | G | -0.2049 | 0.0197 | 0.9706 | 1.92E-25 | 87912 | 335059 | 246405 | 0.0024 | 594 |
| rs9316706 | 13 | 22589883 | A | G | 0.0436 | 0.0067 | 0.3519 | 6.27E-11 | 88109 | 339395 | 247159 | 0.0009 | 214 |
| rs10467680 | 13 | 26780420 | T | C | -0.065 | 0.0065 | 0.434 | 8.53E-24 | 88109 | 339395 | 247159 | 0.0021 | 514 |
| rs12429937 | 13 | 33571753 | A | T | -0.0727 | 0.01 | 0.8772 | 2.99E-13 | 87912 | 335059 | 246405 | 0.0011 | 282 |
| rs123378 | 13 | 51088809 | A | G | -0.0541 | 0.008 | 0.8027 | 1.69E-11 | 88109 | 339395 | 247159 | 0.0009 | 229 |
| rs1215468 | 13 | 80707429 | A | G | 0.0839 | 0.0072 | 0.719 | 1.20E-31 | 88109 | 339395 | 247159 | 0.0028 | 705 |
| rs9583907 | 13 | 91939270 | T | C | -0.0867 | 0.0086 | 0.1691 | 4.27E-24 | 88109 | 339395 | 247159 | 0.0021 | 523 |
| rs12437434 | 14 | 24878370 | T | C | -0.0524 | 0.0072 | 0.2874 | 2.53E-13 | 87912 | 335059 | 246405 | 0.0011 | 278 |
| rs61975988 | 14 | 38809661 | A | G | 0.0376 | 0.0064 | 0.4601 | 3.25E-09 | 88109 | 339395 | 247159 | 0.0007 | 174 |
| rs79823890 | 14 | 52511969 | T | G | -0.0566 | 0.0096 | 0.1332 | 3.34E-09 | 88109 | 339395 | 247159 | 0.0007 | 183 |
| rs2056855 | 14 | 77297139 | T | C | 0.0391 | 0.0067 | 0.3546 | 5.22E-09 | 88109 | 339395 | 247159 | 0.0007 | 173 |
| rs72627178 | 14 | 77372210 | A | G | -0.0505 | 0.0068 | 0.6384 | 8.23E-14 | 85780 | 337056 | 242491 | 0.0012 | 291 |
| rs73347525 | 14 | 101255172 | A | G | 0.0669 | 0.0082 | 0.7579 | 3.20E-16 | 85621 | 335432 | 241911 | 0.0016 | 407 |
| rs55700915 | 14 | 103237952 | A | G | 0.0426 | 0.0066 | 0.4389 | 9.24E-11 | 87912 | 335059 | 246405 | 0.0009 | 221 |
| rs76704029 | 15 | 28546173 | T | C | 0.0464 | 0.0084 | 0.7115 | 3.58E-08 | 77339 | 249112 | 209548 | 0.0009 | 219 |
| rs8043085 | 15 | 38828140 | T | G | 0.0514 | 0.0065 | 0.4495 | 2.14E-15 | 88109 | 339395 | 247159 | 0.0013 | 324 |
| rs3743140 | 15 | 40616742 | A | G | 0.077 | 0.0078 | 0.2331 | 3.71E-23 | 85583 | 332720 | 241737 | 0.0021 | 525 |
| rs149336329 | 15 | 52587740 | T | G | -0.112 | 0.0149 | 0.056 | 5.23E-14 | 87912 | 335059 | 246405 | 0.0013 | 328 |
| rs7161785 | 15 | 62395224 | C | G | -0.0847 | 0.0065 | 0.4154 | 2.71E-39 | 88109 | 339395 | 247159 | 0.0035 | 864 |
| rs4299117 | 15 | 75789444 | T | C | -0.0531 | 0.0065 | 0.3926 | 4.69E-16 | 88109 | 339395 | 247159 | 0.0013 | 333 |
| rs8034133 | 15 | 77763712 | C | G | -0.0848 | 0.0065 | 0.6035 | 5.63E-39 | 88109 | 339395 | 247159 | 0.0034 | 854 |
| rs2290202 | 15 | 91512267 | T | G | 0.0677 | 0.0064 | 0.488 | 6.58E-26 | 88109 | 339395 | 247159 | 0.0023 | 567 |
| rs7167984 | 15 | 93832067 | A | G | -0.047 | 0.0066 | 0.5575 | 7.21E-13 | 88109 | 339395 | 247159 | 0.0011 | 270 |
| rs740862 | 16 | 3689678 | T | G | -0.0384 | 0.0066 | 0.5274 | 4.94E-09 | 88109 | 339395 | 247159 | 0.0007 | 182 |
| rs73541251 | 16 | 20331737 | C | G | -0.0987 | 0.0118 | 0.9209 | 6.84E-17 | 88109 | 339395 | 247159 | 0.0014 | 351 |
| rs11642015 | 16 | 53802494 | T | C | 0.1299 | 0.0085 | 0.1751 | 3.46E-53 | 88109 | 339395 | 247159 | 0.0049 | 1211 |
| rs12051517 | 16 | 72022866 | A | G | -0.0358 | 0.0064 | 0.5687 | 2.15E-08 | 88109 | 339395 | 247159 | 0.0006 | 155 |
| rs6416749 | 16 | 73100308 | T | C | -0.0565 | 0.0069 | 0.6104 | 1.71E-16 | 87753 | 333435 | 245826 | 0.0015 | 376 |
| rs2925979 | 16 | 81534790 | T | C | 0.0422 | 0.0066 | 0.3656 | 1.73E-10 | 88109 | 339395 | 247159 | 0.0008 | 204 |
| rs13342232 | 17 | 6945940 | A | G | -0.1196 | 0.011 | 0.9047 | 1.10E-27 | 88109 | 339395 | 247159 | 0.0025 | 611 |
| rs2905798 | 17 | 29536990 | A | G | 0.0444 | 0.0071 | 0.671 | 2.95E-10 | 88109 | 339395 | 247159 | 0.0009 | 215 |
| rs35551980 | 17 | 36049552 | T | C | -0.0726 | 0.0074 | 0.7469 | 1.02E-22 | 87912 | 335059 | 246405 | 0.0020 | 494 |
| rs11651052 | 17 | 36102381 | A | G | 0.1235 | 0.007 | 0.3058 | 2.46E-69 | 87950 | 337771 | 246579 | 0.0065 | 1611 |
| rs11650046 | 17 | 76777694 | A | C | -0.038 | 0.0065 | 0.4006 | 4.77E-09 | 88109 | 339395 | 247159 | 0.0007 | 172 |
| rs9948462 | 18 | 7076836 | T | C | 0.049 | 0.0071 | 0.7024 | 7.40E-12 | 88109 | 339395 | 247159 | 0.0010 | 248 |
| rs7235574 | 18 | 42368034 | A | G | -0.0419 | 0.0074 | 0.2552 | 1.29E-08 | 88109 | 339395 | 247159 | 0.0007 | 165 |
| rs571312 | 18 | 57839769 | A | C | 0.0887 | 0.0077 | 0.216 | 7.65E-31 | 88109 | 339395 | 247159 | 0.0027 | 660 |
| rs12957245 | 18 | 74614501 | T | C | -0.0382 | 0.0067 | 0.3402 | 1.47E-08 | 87912 | 335059 | 246405 | 0.0007 | 162 |
| rs8101064 | 19 | 7293119 | T | C | 0.0641 | 0.0104 | 0.1264 | 6.47E-10 | 87912 | 335059 | 246405 | 0.0009 | 224 |
| rs2115107 | 19 | 7968168 | A | G | 0.0374 | 0.0068 | 0.3534 | 4.54E-08 | 85780 | 337056 | 242491 | 0.0006 | 158 |
| rs150182828 | 19 | 21856746 | T | C | 0.2367 | 0.0198 | 0.9658 | 8.19E-33 | 87912 | 335059 | 246405 | 0.0037 | 918 |
| rs10422861 | 19 | 33894846 | T | C | -0.0565 | 0.0065 | 0.4636 | 2.06E-18 | 85583 | 332720 | 241737 | 0.0016 | 393 |
| rs58304657 | 19 | 46176405 | C | G | 0.0778 | 0.0065 | 0.514 | 8.29E-33 | 85621 | 335432 | 241911 | 0.0030 | 750 |
| rs6036116 | 20 | 22430350 | T | C | 0.0405 | 0.0066 | 0.6391 | 9.66E-10 | 88109 | 339395 | 247159 | 0.0008 | 187 |
| rs12625671 | 20 | 42994812 | T | C | -0.0673 | 0.0065 | 0.5594 | 2.89E-25 | 88109 | 339395 | 247159 | 0.0022 | 553 |
| rs6122888 | 20 | 48826880 | T | C | 0.0521 | 0.0065 | 0.5086 | 1.31E-15 | 88109 | 339395 | 247159 | 0.0014 | 336 |
| rs7121 | 20 | 57478807 | T | C | 0.0393 | 0.0066 | 0.5972 | 2.52E-09 | 88109 | 339395 | 247159 | 0.0007 | 184 |
| rs5762925 | 22 | 29369398 | A | C | -0.0355 | 0.0064 | 0.5065 | 3.53E-08 | 88109 | 339395 | 247159 | 0.0006 | 156 |
| rs28637892 | 22 | 46313618 | T | G | 0.0563 | 0.0081 | 0.2192 | 3.00E-12 | 80101 | 322056 | 228723 | 0.0011 | 268 |
| rs6520050 | 22 | 46478195 | A | G | 0.0596 | 0.0098 | 0.849 | 1.30E-09 | 85583 | 332720 | 241737 | 0.0009 | 225 |
| rs28645887 | 22 | 50356274 | T | C | -0.0636 | 0.007 | 0.4436 | 6.67E-20 | 85583 | 332720 | 241737 | 0.0020 | 494 |

^a^ Based on hg19, build 37.

Abbreviations: SNP, single nucleotide polymorphism; CHR, chromosome; POS, position; EAF, effective allele frequency.

## Table S4: Baseline characteristics of participants categorised by T2DM status during follow-up^a^

| **Characteristic** | **Men** | | **Women** | |
| --- | --- | --- | --- | --- |
|  | **New-onset T2DM** | **No diabetes at follow-up** | **New-onset T2DM** | **No diabetes at follow-up** |
|  | (N=9,206) | (N=188,450) | (N=14,392) | (N=268,090) |
| Baseline age, years (mean, SD) | 55.0(10.2) | 52.4(10.9) | 55.3(9.5) | 50.7(10.4) |
| Age at T2DM diagnosis (mean, SD) | 62.4(10.2) | .. | 62.6(9.6) | .. |
| **Socioeconomic and lifestyle factors** |  | | | |
| ≥ 6 years of education, N (%) | 5,100(55.4) | 108,560(57.6) | 4,742(32.9) | 118,796(44.3) |
| Annual household income ≥ 35,000 yuan, N (%) | 2,367(25.7) | 37,243(19.8) | 2,723(18.9) | 43,768(16.3) |
| Ever regular smokers^b^, N (%) | 6,874(74.7) | 140,374(74.5) | 716(5.0) | 8,083(3.0) |
| Ever regular alcohol drinkers^c^, N (%) | 3,578(38.9) | 69,664(37.0) | 386(2.7) | 6,763(2.5) |
| Physical activity, MET h/d (mean, SD) | 20.5(14.9) | 22.5(15.3) | 19.1(12.7) | 20.9(12.8) |
| **Anthropometry** |  | | | |
| BMI, kg/m^2^ (mean, SD) | 25.5(3.4) | 23.3(3.2) | 25.8(3.7) | 23.6(3.4) |
| General obese^d^, N (%) | 2,033(22.1) | 14,660(7.8) | 3,725(25.9) | 2,774(10.3) |
| WC, cm (mean, SD) | 87.9(10.0) | 81.4(9.6) | 84.4(9.8) | 78.4(9.3) |
| Central obese^e^, N (%) | 4,015(43.6) | 37,323(19.8) | 6,833(47.5) | 61,988(23.1) |
| **Disease history** |  | | | |
| Cirrhosis/chronic hepatitis, N (%) | 187(2.0) | 3,197(1.7) | 139(1.0) | 2,225(0.8) |

Abbreviations: MET, metabolic equivalent task; BMI, body mass index; WC, waist circumference.

^a^ Regardless of cancer diagnosis. ^b^ Ever regular smokers included former and current regular smokers. ^c^ Ever regular alcohol drinkers included former regular and current weekly drinkers. ^d^ General obesity was defined by a BMI ≥ 28 kg/m^2^. ^e^ Central obesity was defined by a WC ≥ 90 cm in men and ≥ 85 cm in women, respectively.

## Table S5: Characteristics in participants with new-onset T2DM who failed to find any match in 1:3 matching

| **Men (N=37)** | | | | **Women (N=36)** | | | |
| --- | --- | --- | --- | --- | --- | --- | --- |
| **Strata number^a^** | **Study region** | **Baseline body mass index, kg/m^2^** | **Age at T2DM diagnosis, years** | **Strata number^a^** | **Study region** | **Baseline body mass index, kg/m^2^** | **Age at T2DM diagnosis, years** |
| 8375 | *Zhejiang* | 28.6 | 78.9 | 9010 | *Zhejiang* | 30.1 | 78.8 |
| 8685 | *Zhejiang* | 28.9 | 76.4 | 5865 | *Zhejiang* | 31.4 | 78.5 |
| 8611 | *Zhejiang* | 29.9 | 79.3 | 8834 | *Sichuan* | 33.3 | 87.1 |
| 6491 | *Zhejiang* | 30.5 | 74.2 | 5315 | *Zhejiang* | 33.9 | 72.5 |
| 8141 | *Zhejiang* | 30.6 | 69.4 | 11441 | *Sichuan* | 34.3 | 62.8 |
| 8295 | *Zhejiang* | 30.9 | 73.1 | 12463 | *Hunan* | 34.4 | 83.7 |
| 5904 | *Sichuan* | 31.5 | 69.7 | 10237 | *Henan* | 34.6 | 85.4 |
| 8430 | *Sichuan* | 31.6 | 69.6 | 11231 | *Sichuan* | 35.0 | 78.5 |
| 6159 | *Zhejiang* | 31.9 | 64.1 | 401 | *Zhejiang* | 35.2 | 62.7 |
| 7659 | *Sichuan* | 32.0 | 64.4 | 8288 | *Sichuan* | 36.1 | 52.9 |
| 7906 | *Harbin* | 32.9 | 74.0 | 1220 | *Sichuan* | 36.3 | 72.3 |
| 75 | *Zhejiang* | 33.0 | 75.1 | 10713 | *Sichuan* | 36.4 | 70.7 |
| 2513 | *Gansu* | 33.0 | 73.0 | 1431 | *Zhejiang* | 36.6 | 57.6 |
| 7271 | *Liuzhou* | 33.4 | 52.0 | 12776 | *Haikou* | 36.8 | 80.3 |
| 7599 | *Haikou* | 33.4 | 72.4 | 2758 | *Haikou* | 37.2 | 66.4 |
| 1603 | *Gansu* | 33.9 | 47.8 | 12729 | *Haikou* | 37.7 | 46.0 |
| 2194 | *Gansu* | 34.1 | 61.7 | 4084 | *Harbin* | 37.8 | 83.0 |
| 1955 | *Liuzhou* | 34.3 | 80.2 | 6020 | *Zhejiang* | 38.2 | 64.7 |
| 2021 | *Sichuan* | 35.0 | 59.8 | 13198 | *Gansu* | 38.3 | 54.1 |
| 8537 | *Harbin* | 35.2 | 62.2 | 8434 | *Sichuan* | 38.6 | 70.4 |
| 5781 | *Qingdao* | 35.6 | 57.3 | 2433 | *Haikou* | 38.9 | 59.1 |
| 5799 | *Harbin* | 36.0 | 71.0 | 3596 | *Harbin* | 39.2 | 53.3 |
| 3785 | *Harbin* | 36.4 | 69.3 | 11645 | *Hunan* | 39.2 | 50.4 |
| 7190 | *Suzhou* | 36.7 | 53.2 | 12522 | *Henan* | 39.7 | 60.3 |
| 6569 | *Zhejiang* | 37.3 | 70.0 | 3589 | *Harbin* | 40.1 | 62.1 |
| 4622 | *Sichuan* | 37.6 | 72.9 | 4265 | *Sichuan* | 40.2 | 49.2 |
| 5591 | *Henan* | 37.9 | 50.5 | 13051 | *Gansu* | 40.4 | 52.1 |
| 3057 | *Zhejiang* | 38.2 | 50.0 | 8804 | *Suzhou* | 40.6 | 69.7 |
| 6049 | *Sichuan* | 38.2 | 46.8 | 2341 | *Liuzhou* | 41.3 | 52.7 |
| 789 | *Qingdao* | 38.5 | 58.1 | 10319 | *Harbin* | 41.3 | 66.9 |
| 1842 | *Harbin* | 38.8 | 48.7 | 3790 | *Sichuan* | 41.8 | 47.0 |
| 5394 | *Haikou* | 39.3 | 60.4 | 2931 | *Haikou* | 41.9 | 61.1 |
| 3718 | *Zhejiang* | 39.6 | 61.9 | 9462 | *Harbin* | 42.0 | 57.5 |
| 454 | *Liuzhou* | 41.8 | 61.5 | 8363 | *Henan* | 43.3 | 79.2 |
| 6525 | *Sichuan* | 43.3 | 51.6 | 8053 | *Harbin* | 46.2 | 65.4 |
| 8391 | *Henan* | 45.7 | 48.9 | 1666 | *Suzhou* | 51.0 | 58.4 |
| 2962 | *Harbin* | 47.9 | 62.9 | .. | .. | .. | .. |

^a^ Strata number was assigned separately for men and women based on the calendar time of their T2DM diagnosis, following a chronologic order.

## Table S6: Examination in censoring patterns following 1:3 exact matching

| **Event/ censoring** | **Men** | | **Women** | |
| --- | --- | --- | --- | --- |
|  | **New-onset T2DM** | **Matched unexposed** | **New-onset T2DM** | **Matched unexposed** |
|  | (N=8,657) | (N=25,852) | (N=13,680) | (N=40,938) |
| Incident cancer diagnosis, N % | 542(6.3) | 964(3.7) | 582(4.3) | 1262(3.1) |
| Administrative censoring, N % | 7230(83.5) | 22191(85.8) | 12120(88.6) | 36170(88.4) |
| Loss to follow-up, N % | 4(0.05) | 123(0.5) | 13(0.1) | 199(0.5) |
| Non-cancer death^a^, N % | 881(10.2) | 1366(5.3) | 965(7.1) | 1321(3.2) |
| Incident T2DM diagnosis^b^, N % | .. | 1208(4.7) | .. | 1986(4.9) |

^a^ Before a cancer or T2DM diagnosis (in matched controls). ^b^ Before a cancer diagnosis.

## Table S7: Comparison of time-split effect estimates from cause-specific and subdistribution hazard models for T2DM on first primary cancer incidence

| **Outcome** | **Men** | | **Women** | |
| --- | --- | --- | --- | --- |
|  | **Subdistribution hazard model** | **Cause-specific hazard model** | **Subdistribution hazard model** | **Cause-specific hazard model** |
|  | **tsHR (95% CI)** | **tsHR (95% CI)** | **tsHR (95% CI)** | **tsHR (95% CI)** |
| Total first primary cancers | 1.49(1.32–1.69) | 1.57(1.38–1.78) | 1.18(1.06–1.32) | 1.29(1.14–1.46) |
| ORCs | 1.64(1.31–2.04) | 1.73(1.37–2.19) | 1.12(0.94–1.34) | 1.34(1.10–1.62) |
| Total cancers minus ORCs | 1.42(1.22–1.64) | 1.49(1.28–1.74) | 1.22(1.06–1.41) | 1.27(1.09–1.47) |
| Digestive tract organ cancers | 1.27(1.02–1.57) | 1.33(1.06–1.67) | 1.11(0.87–1.41) | 1.14(0.89–1.46) |
| Digestive accessory organ cancers | 2.12(1.56–2.89) | 2.23(1.59–3.11) | 1.79(1.26–2.53) | 2.94(1.94–4.45) |
| ORCs in reproductive organs | .. | .. | 0.88(0.67–1.15) | 0.90(0.68–1.20) |
| Site-specific cancer |  | | | |
| Liver | 2.03(1.40–2.93) | 2.12(1.42–3.15) | 1.64(1.06–2.53) | 2.39(1.42–4.04) |
| Pancreas | 2.34(1.32–4.14) | 2.57(1.35–4.92) | 2.21(1.25–3.89) | 3.95(1.92–8.13) |
| Colorectum | 1.29(0.92–1.81) | 1.33(0.93–1.91) | 1.11(0.79–1.55) | 1.16(0.82–1.64) |
| Lung | 1.45(1.11–1.89) | 1.55(1.17–2.05) | 1.12(0.85–1.46) | 1.15(0.87–1.52) |
| Stomach | 1.08(0.75–1.56) | 1.13(0.77–1.68) | 1.11(0.72–1.69) | 1.11(0.72–1.71) |
| Breast (overall) | .. | .. | 0.98(0.73–1.33) | 1.01(0.73–1.38) |
| Postmenopausal breast | .. | .. | 0.96(0.70–1.31) | 1.02(0.74–1.41) |

Abbreviations: ORCs, obesity-related cancers; tsHR, time-split hazard ratio.

Each cell contains the tsHR and associated 95% CI for the given outcome and for the follow-up time window beyond 12 months in new-onset T2DM compared to the matched unexposed following 1:3 exact matching. All models were stratified on the matched set and further adjusted for waist circumference, smoking status, alcohol consumption habit, MET hours, highest education level, annual household income, and cirrhosis/chronic hepatitis history (for liver cancer and for composite cancer outcomes that encompassed liver cancer).

## Table S8: Associations of new-onset T2DM with composite cancer risks after applying a narrower caliper width in BMI matching

|  | | **Men** | | **Women** | |
| --- | --- | --- | --- | --- | --- |
|  |  | **New-onset T2DM** | **Matched unexposed** | **New-onset T2DM** | **Matched unexposed** |
| No. of participants | | 8,610 | 25,627 | 13,642 | 40,700 |
| No. of person-years | | 39,493 | 119,350 | 67,123 | 199,269 |
| *Total first primary cancers* | |  |  |  |  |
| No. of cases | | 539 | 1,001 | 580 | 1,243 |
| IR^a^ | | 1364.8(1249.6–1480.0) | 838.7(786.8–890.7) | 864.1(793.8–934.4) | 623.8(589.1–658.5) |
| tsHR (95% CI)^b^ | Model 1^c^ | 1.43(1.26–1.62) | 1(ref) | 1.33(1.19–1.50) | 1(ref) |
|  | Model 2^d^ | 1.40(1.24–1.59) | 1(ref) | 1.33(1.18–1.49) | 1(ref) |
| *ORCs^e^* | |  |  |  |  |
| No. of cases | | 175 | 284 | 220 | 475 |
| IR^a^ | | 443.1(377.5–508.8) | 238.0(210.3–265.6) | 327.8(284.4–371.1) | 238.4(216.9–259.8) |
| tsHR (95% CI)^b^ | Model 1^c^ | 1.64(1.31–2.06) | 1(ref) | 1.44(1.18–1.74) | 1(ref) |
|  | Model 2^d^ | 1.60(1.27–2.02) | 1(ref) | 1.44(1.18–1.75) | 1(ref) |
| *Total cancers minus ORCs* | |  |  |  |  |
| No. of cases | | 364 | 717 | 360 | 768 |
| IR^a^ | | 921.7(827.0–1016.4) | 600.8(556.8–644.7) | 536.3(480.9–591.7) | 385.4(358.2–412.7) |
| tsHR (95% CI)^b^ | Model 1^c^ | 1.34(1.16–1.56) | 1(ref) | 1.33(1.15–1.54) | 1(ref) |
|  | Model 2^d^ | 1.33(1.14–1.55) | 1(ref) | 1.32(1.14–1.53) | 1(ref) |
| *Digestive tract organ cancers^f^* | |  |  |  |  |
| No. of cases | | 160 | 353 | 127 | 327 |
| IR^a^ | | 405.1(342.4–467.9) | 295.8(264.9–326.6) | 189.2(156.3–222.1) | 164.1(146.3–181.9) |
| tsHR (95% CI)^b^ | Model 1^c^ | 1.22(0.98–1.53) | 1(ref) | 1.08(0.85–1.38) | 1(ref) |
|  | Model 2^d^ | 1.23(0.98–1.54) | 1(ref) | 1.07(0.84–1.36) | 1(ref) |
| *Digestive accessory organ cancers^g^* | |  |  |  |  |
| No. of cases | | 103 | 134 | 74 | 91 |
| IR^a^ | | 260.8(210.4–311.2) | 112.3(93.3–131.3) | 110.2(85.1–135.4) | 45.7(36.3–55.0) |
| tsHR (95% CI)^b^ | Model 1^c^ | 1.94(1.42–2.65) | 1(ref) | 2.43(1.67–3.55) | 1(ref) |
|  | Model 2^d^ | 1.87(1.35–2.58) | 1(ref) | 2.45(1.66–3.60) | 1(ref) |
| *ORCs in reproductive organs^h^* | |  |  |  |  |
| No. of cases | | .. | .. | 78 | 223 |
| IR^a^ | | .. | .. | 116.2(90.4–142.0) | 111.9(97.2–126.6) |
| tsHR (95% CI)^b^ | Model 1^c^ | .. | .. | 1.09(0.82–1.46) | 1(ref) |
|  | Model 2^d^ | .. | .. | 1.12(0.84–1.50) | 1(ref) |

Abbreviations: ORCs, obesity-related cancers; IR, incidence rate; tsHR, time-split hazard ratio.

^a^ Per 100,000 person-years. ^b^ The tsHR for follow-up time window over 12 months was presented. ^c^ Model 1 was stratified on the matched set. ^d^ Model 2 was stratified on the matched set and further adjusted for waist circumference, smoking status, alcohol consumption habit, physical activity, highest education level, annual household income, and cirrhosis/chronic hepatitis history (for composite cancer risks that encompassed liver cancer). ^e^ ORCs include cancers of the colon and rectum, liver, pancreas, and (in women) postmenopausal breast, endometrium, and ovary. ^f^ Digestive tract organ cancers include cancers of the oesophagus, stomach, colon and rectum. ^g^ Digestive accessory organ cancers refer to combined liver and pancreatic cancers. ^h^ ORCs in reproductive organs (women only) include postmenopausal breast, endometrial, and ovarian cancers.

Table S9: Associations of new-onset T2DM with site-specific cancer risks after applying a narrower caliper width in BMI matching

|  | | **Men** | | **Women** | |
| --- | --- | --- | --- | --- | --- |
|  |  | **New-onset T2DM** | **Matched unexposed** | **New-onset T2DM** | **Matched unexposed** |
| No. of participants | | 8,610 | 25,627 | 13,642 | 40,700 |
| No. of person-years | | 39,493 | 119,350 | 67,123 | 199,269 |
| *Liver* | |  |  |  |  |
| No. of cases | | 67 | 98 | 42 | 52 |
| IR^a^ | | 169.7(129.0–210.3) | 82.1(65.9–98.4) | 62.6(43.6–81.5) | 26.1(19.0–33.2) |
| tsHR (95% CI)^b^ | Model 1^c^ | 1.83(1.27–2.64) | 1(ref) | 2.32(1.44–3.71) | 1(ref) |
|  | Model 2^d^ | 1.69(1.15–2.50) | 1(ref) | 2.34(1.44–3.81) | 1(ref) |
| *Pancreas* | |  |  |  |  |
| No. of cases | | 36 | 36 | 33 | 39 |
| IR^a^ | | 91.2(61.4–120.9) | 30.2(20.3–40.0) | 49.2(32.4–65.9) | 19.6(13.4–25.7) |
| tsHR (95% CI)^b^ | Model 1^c^ | 2.27(1.27–4.07) | 1(ref) | 1.88(1.08–3.28) | 1(ref) |
|  | Model 2^d^ | 2.50(1.33–4.72) | 1(ref) | 1.84(1.02–3.30) | 1(ref) |
| *Colorectum* | |  |  |  |  |
| No. of cases | | 62 | 139 | 64 | 151 |
| IR^a^ | | 157.0(117.9–196.1) | 116.5(97.1–135.8) | 95.3(72.0–118.7) | 75.8(63.7–87.9) |
| tsHR (95% CI)^b^ | Model 1^c^ | 1.35(0.96–1.91) | 1(ref) | 1.21(0.86–1.71) | 1(ref) |
|  | Model 2^d^ | 1.34(0.94–1.90) | 1(ref) | 1.21(0.85–1.71) | 1(ref) |
| *Lung* | |  |  |  |  |
| No. of cases | | 115 | 236 | 101 | 211 |
| IR^a^ | | 291.2(238.0–344.4) | 197.7(172.5–223.0) | 150.5(121.1–179.8) | 105.9(91.6–120.2) |
| tsHR (95% CI)^b^ | Model 1^c^ | 1.30(1.00–1.70) | 1(ref) | 1.30(0.98–1.72) | 1(ref) |
|  | Model 2^d^ | 1.33(1.01–1.75) | 1(ref) | 1.27(0.95–1.68) | 1(ref) |
| *Stomach* | |  |  |  |  |
| No. of cases | | 59 | 132 | 40 | 106 |
| IR^a^ | | 149.4(111.3–187.5) | 110.6(91.7–129.5) | 59.6(41.1–78.1) | 53.2(43.1–63.3) |
| tsHR (95% CI)^b^ | Model 1^c^ | 0.99(0.68–1.46) | 1(ref) | 0.92(0.61–1.39) | 1(ref) |
|  | Model 2^d^ | 1.02(0.69–1.51) | 1(ref) | 0.92(0.61–1.40) | 1(ref) |
| *Breast (overall)* | |  |  |  |  |
| No. of cases | | .. | .. | 66 | 164 |
| IR^a^ | | .. | .. | 98.3(74.6–122.0) | 82.3(69.7–94.9) |
| tsHR (95% CI)^b^ | Model 1^c^ | .. | .. | 1.23(0.89–1.69) | 1(ref) |
|  | Model 2^d^ | .. | .. | 1.26(0.91–1.74) | 1(ref) |
| *Postmenopausal breast* | |  |  |  |  |
| No. of cases | | .. | .. | 61 | 153 |
| IR^a^ | | .. | .. | 90.9(68.1–113.7) | 76.8(64.6–88.9) |
| tsHR (95% CI)^b^ | Model 1^c^ | .. | .. | 1.23(0.89–1.72) | 1(ref) |
|  | Model 2^d^ | .. | .. | 1.27(0.91–1.77) | 1(ref) |

Abbreviations: IR, incidence rate; tsHR, time-split hazard ratio.

^a^ Per 100,000 person-years. ^b^ The tsHR for follow-up time window over 12 months was presented. ^c^ Model 1 was stratified on the matched set. ^d^ Model 2 was stratified on the matched set and further adjusted for waist circumference, smoking status, alcohol consumption habit, physical activity, highest education level, annual household income, and cirrhosis/chronic hepatitis history (for liver cancer).

## Table S10: Associations of new-onset T2DM with composite cancer risks following 1:5 exact matching

|  | | **Men** | | **Women** | |
| --- | --- | --- | --- | --- | --- |
|  |  | **New-onset T2DM** | **Matched unexposed** | **New-onset T2DM** | **Matched unexposed** |
| No. of participants | | 8,568 | 42,326 | 13,586 | 67,365 |
| No. of person-years | | 39,539 | 199,595 | 67,108 | 331,540 |
| *Total first primary cancers* | |  |  |  |  |
| No. of cases | | 540 | 1657 | 577 | 2076 |
| IR^a^ | | 1365.7(1250.5–1480.9) | 830.2(790.2–870.2) | 859.8(789.7–930.0) | 626.2(599.2–653.1) |
| tsHR (95% CI)^b^ | Model 1^c^ | 1.52(1.35–1.70) | 1(ref) | 1.28(1.16–1.43) | 1(ref) |
|  | Model 2^d^ | 1.50(1.34–1.69) | 1(ref) | 1.27(1.14–1.42) | 1(ref) |
| *ORCs^e^* | |  |  |  |  |
| No. of cases | | 176 | 441 | 219 | 846 |
| IR^a^ | | 445.1(379.4–510.9) | 220.9(200.3–241.6) | 326.3(283.1–369.6) | 255.2(238.0–272.4) |
| tsHR (95% CI)^b^ | Model 1^c^ | 1.78(1.44–2.19) | 1(ref) | 1.36(1.13–1.62) | 1(ref) |
|  | Model 2^d^ | 1.76(1.42–2.17) | 1(ref) | 1.34(1.12–1.60) | 1(ref) |
| *Total cancers minus ORCs* | |  |  |  |  |
| No. of cases | | 364 | 1216 | 358 | 1230 |
| IR^a^ | | 920.6(826.0–1015.2) | 609.2(575.0–643.5) | 533.5(478.2–588.7) | 371.0(350.3–391.7) |
| tsHR (95% CI)^b^ | Model 1^c^ | 1.42(1.23–1.63) | 1(ref) | 1.34(1.17–1.54) | 1(ref) |
|  | Model 2^d^ | 1.41(1.22–1.62) | 1(ref) | 1.33(1.16–1.52) | 1(ref) |
| *Digestive tract organ cancers^f^* | |  |  |  |  |
| No. of cases | | 161 | 599 | 124 | 518 |
| IR^a^ | | 407.2(344.3–470.1) | 300.1(276.1–324.1) | 184.8(152.3–217.3) | 156.2(142.8–169.7) |
| tsHR (95% CI)^b^ | Model 1^c^ | 1.29(1.05–1.58) | 1(ref) | 1.12(0.89–1.41) | 1(ref) |
|  | Model 2^d^ | 1.31(1.07–1.61) | 1(ref) | 1.10(0.87–1.38) | 1(ref) |
| *Digestive accessory organ cancers^g^* | |  |  |  |  |
| No. of cases | | 104 | 183 | 74 | 172 |
| IR^a^ | | 263.0(212.5–313.6) | 91.7(78.4–105.0) | 110.3(85.1–135.4) | 51.9(44.1–59.6) |
| tsHR (95% CI)^b^ | Model 1^c^ | 2.60(1.94–3.49) | 1(ref) | 2.11(1.51–2.95) | 1(ref) |
|  | Model 2^d^ | 2.46(1.81–3.35) | 1(ref) | 2.12(1.51–2.97) | 1(ref) |
| *ORCs in reproductive organs^h^* | |  |  |  |  |
| No. of cases | | .. | .. | 79 | 387 |
| IR^a^ | | .. | .. | 117.7(91.8–143.7) | 116.7(105.1–128.4) |
| tsHR (95% CI)^b^ | Model 1^c^ | .. | .. | 1.04(0.79–1.35) | 1(ref) |
|  | Model 2^d^ | .. | .. | 1.03(0.79–1.35) | 1(ref) |

Abbreviations: ORCs, obesity-related cancers; IR, incidence rate; tsHR, time-split hazard ratio.

^a^ Per 100,000 person-years. ^b^ The tsHR for follow-up time window over 12 months was presented. ^c^ Model 1 was stratified on the matched set. ^d^ Model 2 was stratified on the matched set and further adjusted for waist circumference, smoking status, alcohol consumption habit, physical activity, highest education level, annual household income, and cirrhosis/chronic hepatitis history (for composite cancer risks that encompassed liver cancer). ^e^ ORCs include cancers of the colon and rectum, liver, pancreas, and (in women) postmenopausal breast, endometrium, and ovary. ^f^ Digestive tract organ cancers include cancers of the oesophagus, stomach, colon and rectum. ^g^ Digestive accessory organ cancers refer to combined liver and pancreatic cancers. ^h^ ORCs in reproductive organs (women only) include postmenopausal breast, endometrial, and ovarian cancers.

## Table S11: Associations of new-onset T2DM with site-specific cancer risks following 1:5 exact matching

|  | | **Men** | | **Women** | |
| --- | --- | --- | --- | --- | --- |
|  |  | **New-onset T2DM** | **Matched unexposed** | **New-onset T2DM** | **Matched unexposed** |
| No. of participants | | 8,568 | 42,326 | 13,586 | 67,365 |
| No. of person-years | | 39,539 | 199,595 | 67,108 | 331,540 |
| *Liver* | |  |  |  |  |
| No. of cases | | 68 | 137 | 42 | 125 |
| IR^a^ | | 172.0(131.1–212.9) | 68.6(57.1–80.1) | 62.6(43.7–81.5) | 37.7(31.1–44.3) |
| tsHR (95% CI)^b^ | Model 1^c^ | 2.47(1.75–3.50) | 1(ref) | 1.83(1.21–2.78) | 1(ref) |
|  | Model 2^d^ | 2.32(1.61–3.34) | 1(ref) | 1.85(1.21–2.83) | 1(ref) |
| *Pancreas* | |  |  |  |  |
| No. of cases | | 36 | 47 | 33 | 48 |
| IR^a^ | | 91.0(61.3–120.8) | 23.5(16.8–30.3) | 49.2(32.4–66.0) | 14.5(10.4–18.6) |
| tsHR (95% CI)^b^ | Model 1^c^ | 2.99(1.71–5.23) | 1(ref) | 2.44(1.43–4.19) | 1(ref) |
|  | Model 2^d^ | 3.71(2.03–6.81) | 1(ref) | 2.45(1.41–4.25) | 1(ref) |
| *Colorectum* | |  |  |  |  |
| No. of cases | | 62 | 226 | 62 | 267 |
| IR^a^ | | 156.8(117.8–195.8) | 113.2(98.5–128.0) | 92.4(69.4–115.4) | 80.5(70.9–90.2) |
| tsHR (95% CI)^b^ | Model 1^c^ | 1.32(0.96–1.82) | 1(ref) | 1.06(0.77–1.45) | 1(ref) |
|  | Model 2^d^ | 1.31(0.95–1.80) | 1(ref) | 1.03(0.75–1.42) | 1(ref) |
| *Lung* | |  |  |  |  |
| No. of cases | | 115 | 348 | 101 | 338 |
| IR^a^ | | 290.9(237.7–344.0) | 174.4(156.0–192.7) | 150.5(121.2–179.9) | 101.9(91.1–112.8) |
| tsHR (95% CI)^b^ | Model 1^c^ | 1.53(1.19–1.97) | 1(ref) | 1.37(1.06–1.77) | 1(ref) |
|  | Model 2^d^ | 1.52(1.17–1.96) | 1(ref) | 1.33(1.02–1.73) | 1(ref) |
| *Stomach* | |  |  |  |  |
| No. of cases | | 60 | 224 | 39 | 151 |
| IR^a^ | | 151.7(113.4–190.1) | 112.2(97.5–126.9) | 58.1(39.9–76.4) | 45.5(38.3–52.8) |
| tsHR (95% CI)^b^ | Model 1^c^ | 1.13(0.79–1.62) | 1(ref) | 1.32(0.87–2.00) | 1(ref) |
|  | Model 2^d^ | 1.19(0.83–1.71) | 1(ref) | 1.30(0.86–1.98) | 1(ref) |
| *Breast (overall)* | |  |  |  |  |
| No. of cases | | .. | .. | 68 | 301 |
| IR^a^ | | .. | .. | 101.3(77.2–125.4) | 90.8(80.5–101.0) |
| tsHR (95% CI)^b^ | Model 1^c^ | .. | .. | 1.11(0.83–1.49) | 1(ref) |
|  | Model 2^d^ | .. | .. | 1.12(0.83–1.50) | 1(ref) |
| *Postmenopausal breast* | |  |  |  |  |
| No. of cases | | .. | .. | 62 | 286 |
| IR^a^ | | .. | .. | 92.4(69.4–115.4) | 86.3(76.3–96.3) |
| tsHR (95% CI)^b^ | Model 1^c^ | .. | .. | 1.09(0.81–1.48) | 1(ref) |
|  | Model 2^d^ | .. | .. | 1.10(0.81–1.49) | 1(ref) |

Abbreviations: IR, incidence rate; tsHR, time-split hazard ratio.

^a^ Per 100,000 person-years. ^b^ The tsHR for follow-up time window over 12 months was presented. ^c^ Model 1 was stratified on the matched set. ^d^ Model 2 was stratified on the matched set and further adjusted for waist circumference, smoking status, alcohol consumption habit, physical activity, highest education level, annual household income, and cirrhosis/chronic hepatitis history (for liver cancer).

## Table S12: Associations of new-onset T2DM with composite cancer risks in smoking- and alcohol-naïve women

|  | | **Smoking- and alcohol-naïve women** | |
| --- | --- | --- | --- |
|  |  | **New-onset T2DM** | **Matched unexposed** |
| No. of participants | | 9,052 | 27,065 |
| No. of person-years | | 46,172 | 136,658 |
| *Total first primary cancers* | |  |  |
| No. of cases | | 387 | 830 |
| IR^a^ | | 838.2(754.7–921.7) | 607.4(566.0–648.7) |
| tsHR (95% CI)^b^ | Model 1^c^ | 1.29(1.13–1.48) | 1(ref) |
|  | Model 2^d^ | 1.27(1.10–1.46) | 1(ref) |
| *ORCs^e^* | |  |  |
| No. of cases | | 147 | 330 |
| IR^a^ | | 318.4(266.9–369.8) | 241.5(215.4–267.5) |
| tsHR (95% CI)^b^ | Model 1^c^ | 1.42(1.13–1.79) | 1(ref) |
|  | Model 2^d^ | 1.39(1.10–1.76) | 1(ref) |
| *Total cancers minus ORCs* | |  |  |
| No. of cases | | 240 | 500 |
| IR^a^ | | 519.8(454.0–585.6) | 365.9(333.8–397.9) |
| tsHR (95% CI)^b^ | Model 1^c^ | 1.29(1.08–1.55) | 1(ref) |
|  | Model 2^d^ | 1.28(1.07–1.53) | 1(ref) |
| *Digestive tract organ cancers^f^* | |  |  |
| No. of cases | | 86 | 212 |
| IR ^a^ | | 186.3(146.9–225.6) | 155.1(134.2–176.0) |
| tsHR (95% CI) ^b^ | Model 1^c^ | 1.07(0.79–1.44) | 1(ref) |
|  | Model 2^d^ | 1.03(0.76–1.39) | 1(ref) |
| *Digestive accessory organ cancers^g^* | |  |  |
| No. of cases | | 50 | 71 |
| IR^a^ | | 108.3(78.3–138.3) | 52.0(39.9–64.0) |
| tsHR (95% CI)^b^ | Model 1^c^ | 1.92(1.27–2.88) | 1(ref) |
|  | Model 2^d^ | 2.05(1.34–3.12) | 1(ref) |
| *ORCs in reproductive organs^h^* | |  |  |
| No. of cases | | 47 | 140 |
| IR^a^ | | 101.8(72.7–130.9) | 102.4(85.5–119.4) |
| tsHR (95% CI)^b^ | Model 1^c^ | 1.12(0.78–1.61) | 1(ref) |
|  | Model 2^d^ | 1.10(0.76–1.59) | 1(ref) |

Abbreviations: ORCs, obesity-related cancers; IR, incidence rate; tsHR, time-split hazard ratio.

^a^ Per 100,000 person-years. ^b^ The tsHR for follow-up time window over 12 months was presented. ^c^ Model 1 was stratified on the matched set. ^d^ Model 2 was stratified on the matched set and further adjusted for waist circumference, physical activity, highest education level, annual household income, and cirrhosis/chronic hepatitis history (for composite cancer risks that encompassed liver cancer). ^e^ ORCs include cancers of the colon and rectum, liver, pancreas, postmenopausal breast, endometrium, and ovary. ^f^ Digestive tract organ cancers include cancers of the oesophagus, stomach, colon and rectum. ^g^ Digestive accessory organ cancers refer to combined liver and pancreatic cancers. ^h^ ORCs in reproductive organs include postmenopausal breast, endometrial, and ovarian cancers.

## Table S13: Associations of new-onset T2DM with site-specific cancer risks in smoking- and alcohol-naïve women

|  | | **Smoking- and alcohol-naïve women** | |
| --- | --- | --- | --- |
|  |  | **New-onset T2DM** | **Matched unexposed** |
| No. of participants | | 9,052 | 27,065 |
| No. of person-years | | 46,172 | 136,658 |
| *Liver* | |  |  |
| No. of cases | | 30 | 52 |
| IR^a^ | | 65.0(41.7–88.2) | 38.1(27.7–48.4) |
| tsHR (95% CI)^b^ | Model 1^c^ | 1.68(1.02–2.78) | 1(ref) |
|  | Model 2^d^ | 1.83(1.08–3.12) | 1(ref) |
| *Colorectum* | |  |  |
| No. of cases | | 47 | 107 |
| IR^a^ | | 101.8(72.7–130.9) | 78.3(63.5–93.1) |
| tsHR (95% CI)^b^ | Model 1^c^ | 1.11(0.74–1.66) | 1(ref) |
|  | Model 2^d^ | 1.06(0.70–1.60) | 1(ref) |
| *Lung* | |  |  |
| No. of cases | | 61 | 116 |
| IR^a^ | | 132.1(99.0–165.3) | 84.9(69.4–100.3) |
| tsHR (95% CI)^b^ | Model 1^c^ | 1.30(0.91–1.87) | 1(ref) |
|  | Model 2^d^ | 1.26(0.87–1.82) | 1(ref) |
| *Stomach* | |  |  |
| No. of cases | | 30 | 67 |
| IR^a^ | | 65.0(41.7–88.2) | 49.0(37.3–60.8) |
| tsHR (95% CI)^b^ | Model 1^c^ | 1.13(0.67–1.91) | 1(ref) |
|  | Model 2^d^ | 1.07(0.63–1.82) | 1(ref) |
| *Breast (overall)* | |  |  |
| No. of cases | | 40 | 102 |
| IR^a^ | | 86.6(59.8–113.5) | 74.6(60.2–89.1) |
| tsHR (95% CI)^b^ | Model 1^c^ | 1.36(0.92–2.02) | 1(ref) |
|  | Model 2^d^ | 1.31(0.88–1.97) | 1(ref) |
| *Postmenopausal breast* | |  |  |
| No. of cases | | 37 | 97 |
| IR^a^ | | 80.1(54.3–106.0) | 71.0(56.9–85.1) |
| tsHR (95% CI)^b^ | Model 1^c^ | 1.31(0.87–1.98) | 1(ref) |
|  | Model 2^d^ | 1.28(0.84–1.94) | 1(ref) |

Abbreviations: IR, incidence rate; tsHR, time-split hazard ratio.

^a^ Per 100,000 person-years. ^b^ The tsHR for follow-up time window over 12 months was presented. ^c^ Model 1 was stratified on the matched set. ^d^ Model 2 was stratified on the matched set and further adjusted for waist circumference, physical activity, highest education level, annual household income, and cirrhosis/chronic hepatitis history (for liver cancer).

## Table S14: MR sensitivity analyses of the associations between T2DM and major site-specific cancers in East Asians

| **Cancer site & Source (No. of SNPs)** | **OR (95%CI)^a^** | **P** |
| --- | --- | --- |
| Colorectum & BBJ-CRC (183 SNPs) |  |  |
| IVW (P for heterogeneity=0.02) | 1.01 (0.97–1.05) | 0.62 |
| Weighted median | 0.97 (0.91–1.02) | 0.25 |
| Weighted mode | 0.95 (0.89–1.02) | 0.12 |
| MR-Egger (P for pleiotropy=0.13) | 0.96 (0.89–1.03) | 0.28 |
| MR-PRESSO (no outliers detected) | 1.01 (0.97–1.05) | 0.62 |
| Colorectum & CKB (185 SNPs) |  |  |
| IVW (P for heterogeneity=0.20) | 0.97(0.90–1.05) | 0.50 |
| Weighted median | 0.98(0.85–1.13) | 0.77 |
| Weighted mode | 0.96(0.80–1.15) | 0.67 |
| MR-Egger (P for pleiotropy=0.80) | 0.95(0.81–1.12) | 0.58 |
| MR-PRESSO (no outliers detected) | 0.97(0.90–1.05) | 0.50 |
| Pancreas & JaPAN+NCC+BBJ-PaC (170 SNPs) |  |  |
| IVW (P for heterogeneity=0.64) | 1.10(1.03–1.17) | 0.0033 |
| Weighted median | 1.13(1.02–1.26) | 0.03 |
| Weighted mode | 1.16(1.04–1.30) | 0.02 |
| MR-Egger (P for pleiotropy=0.47) | 1.14(1.01–1.30) | 0.03 |
| MR-PRESSO (no outliers detected) | 1.10(1.03–1.17) | 0.0033 |
| Pancreas & CKB (185 SNPs) |  |  |
| IVW (P for heterogeneity=0.65) | 0.97(0.82–1.14) | 0.67 |
| Weighted median | 0.90(0.68–1.18) | 0.44 |
| Weighted mode | 0.81(0.57–1.15) | 0.24 |
| MR-Egger (P for pleiotropy=0.14) | 0.78(0.57–1.08) | 0.14 |
| MR-PRESSO (no outliers detected) | 0.97(0.82–1.14) | 0.67 |
| Breast & KoGES (182 SNPs) |  |  |
| IVW (P for heterogeneity=0.04) | 1.05(0.93–1.19) | 0.40 |
| Weighted median | 1.09(0.91–1.32) | 0.35 |
| Weighted mode | 1.09(0.87–1.37) | 0.44 |
| MR-Egger (P for pleiotropy=0.02) | 0.82(0.64–1.04) | 0.10 |
| MR-PRESSO (no outliers detected) | 1.05(0.93–1.19) | 0.40 |
| Breast & CKB (185 SNPs) |  |  |
| IVW (P for heterogeneity= 0.79) | 1.04(0.95–1.14) | 0.37 |
| Weighted median | 1.08(0.93–1.25) | 0.36 |
| Weighted mode | 1.10(0.90–1.34) | 0.35 |
| MR-Egger (P for pleiotropy=0.50) | 0.99(0.82–1.19) | 0.89 |
| MR-PRESSO (no outliers detected) | 1.04(0.95–1.14) | 0.37 |
| Endometrium & KoGES (182 SNPs) |  |  |
| IVW (P for heterogeneity=0.31) | 0.97(0.86–1.10) | 0.62 |
| Weighted median | 1.08(0.87–1.34) | 0.47 |
| Weighted mode | 1.07(0.84–1.36) | 0.61 |
| MR-Egger (P for pleiotropy=0.42) | 1.06(0.83–1.36) | 0.65 |
| MR-PRESSO (no outliers detected) | 0.97(0.86–1.10) | 0.62 |
| Endometrium & CKB (185 SNPs) |  |  |
| IVW (P for heterogeneity=0.96) | 1.15(0.91–1.45) | 0.25 |
| Weighted median | 1.33(0.90–1.98) | 0.15 |
| Weighted mode | .. | .. |
| MR-Egger (P for pleiotropy=0.50) | 1.32(0.83–2.11) | 0.25 |
| MR-PRESSO (no outliers detected) | 1.15(0.91–1.45) | 0.25 |
| Liver & CKB (185 SNPs) |  |  |
| IVW (P for heterogeneity=0.06) | 1.00(0.91–1.09) | 0.97 |
| Weighted median | 1.10(0.94–1.28) | 0.21 |
| Weighted mode | 1.13(0.94–1.37) | 0.23 |
| MR-Egger (P for pleiotropy=0.44) | 1.06(0.88–1.28) | 0.52 |
| MR-PRESSO (no outliers detected) | 1.00(0.91–1.09) | 0.97 |
| Lung & CKB (185 SNPs) |  |  |
| IVW (P for heterogeneity=0.04) | 1.02(0.96–1.08) | 0.63 |
| Weighted median | 1.05(0.96–1.15) | 0.32 |
| Weighted mode | 1.06(0.95–1.17) | 0.32 |
| MR-Egger (P for pleiotropy=0.85) | 1.03(0.91–1.16) | 0.69 |
| MR-PRESSO (no outliers detected) | 1.02(0.96–1.08) | 0.63 |
| Stomach & CKB (185 SNPs) |  |  |
| IVW (P for heterogeneity=0.76) | 1.00(0.92–1.08) | 0.99 |
| Weighted median | 0.90(0.79–1.03) | 0.13 |
| Weighted mode | 0.91(0.79–1.06) | 0.23 |
| MR-Egger (P for pleiotropy=0.93) | 0.99(0.85–1.17) | 0.94 |
| MR-PRESSO (no outliers detected) | 1.00(0.92–1.08) | 0.99 |

Abbreviations: T2DM, type 2 diabetes mellitus; BBJ-CRC, Biobank Japan colorectal cancer cohort; CKB, China Kadoorie Biobank; JaPAN, Japan pancreatic cancer research; NCC, national cancer centre; BBJ-PaC, Biobank Japan pancreatic cancer cohort; KoGES, Korean genome and epidemiology study; OR, odds ratio; SNPs, single nucleotide polymorphisms.

^a^ Estimates indicating OR of cancer per doubling in odds of T2DM.

## Table S15: Sanderson–Windmeijer conditional F-statistics for exposures included in multivariable Mendelian Randomisation analyses

| **Cancer site** | **Source of outcome summary statistics** | **No. of SNPs^a^** | **Conditional F-statistic for T2DM** | **Conditional F-statistic for BMI** |
| --- | --- | --- | --- | --- |
| Colorectum | BBJ-CRC | 149 | 79.1 | 2.6 |
|  | CKB | 149 | 79.1 | 2.6 |
| Pancreas | JaPAN+NCC+BBJ-PaC | 146 | 81.2 | 2.7 |
|  | CKB | 149 | 79.1 | 2.6 |
| Breast | KoGES | 148 | 80.1 | 2.6 |
|  | CKB | 149 | 79.1 | 2.6 |
| Endometrium | KoGES | 148 | 80.1 | 2.6 |
|  | CKB | 149 | 79.1 | 2.6 |
| Liver | CKB | 149 | 79.1 | 2.6 |
| Lung | CKB | 149 | 79.1 | 2.6 |
| Stomach | CKB | 149 | 79.1 | 2.6 |

Abbreviations: T2DM, type 2 diabetes mellitus; BBJ-CRC, Biobank Japan colorectal cancer cohort; CKB, China Kadoorie Biobank; JaPAN, Japan pancreatic cancer research; NCC, national cancer centre; BBJ-PaC, Biobank Japan pancreatic cancer cohort; KoGES, Korean genome and epidemiology study; SNPs, single nucleotide polymorphisms.

^a^ The number of SNPs varies slightly across outcomes because SNPs unavailable in the corresponding outcome GWAS datasets were excluded during harmonisation.

## Table S16: Power calculation for two-sample Mendelian Randomisation of the associations between T2DM and site-specific cancers^a^

| **Cancer site& source** | **Sample size** | **No. of case** | **No. of control** | **Case-control ratio** | **Odds ratio^b^** | | |
| --- | --- | --- | --- | --- | --- | --- | --- |
|  |  |  |  |  | **0.90** | **1.10** | **1.20** |
| Colorectum |  |  |  |  |  |  |  |
| BBJ-CRC | 33,870 | 6,692 | 27,178 | 4.06 | 1.00 | 0.99 | 1.00 |
| CKB | 100,640 | 912 | 99,728 | 109.35 | 0.51 | 0.43 | 0.93 |
| Pancreas |  |  |  |  |  |  |  |
| JaPAN+NCC+BBJ-PaC | 34,631 | 2,039 | 32,592 | 15.98 | 0.82 | 0.74 | 1.00 |
| CKB | 100,640 | 205 | 100,435 | 489.93 | 0.15 | 0.13 | 0.37 |
| Breast |  |  |  |  |  |  |  |
| KoGES | 46,330 | 424 | 45,906 | 108.27 | 0.27 | 0.23 | 0.65 |
| CKB | 57,625 | 655 | 56,970 | 86.98 | 0.39 | 0.33 | 0.83 |
| Endometrium |  |  |  |  |  |  |  |
| KoGES | 46,330 | 355 | 45,975 | 129.51 | 0.23 | 0.20 | 0.57 |
| CKB | 57,625 | 102 | 57,523 | 563.95 | 0.10 | 0.09 | 0.21 |
| Liver |  |  |  |  |  |  |  |
| CKB | 100,640 | 770 | 99,870 | 129.70 | 0.44 | 0.38 | 0.88 |
| Lung |  |  |  |  |  |  |  |
| CKB | 100,640 | 1,802 | 98,838 | 54.85 | 0.79 | 0.71 | 1.00 |
| Stomach |  |  |  |  |  |  |  |
| CKB | 100,640 | 888 | 99,752 | 112.33 | 0.50 | 0.42 | 0.92 |

Abbreviations: T2DM, type 2 diabetes mellitus; BBJ-CRC, Biobank Japan colorectal cancer cohort; CKB, China Kadoorie Biobank; JaPAN, Japan pancreatic cancer research; NCC, national cancer centre; BBJ-PaC, Biobank Japan pancreatic cancer cohort; KoGES, Korean genome and epidemiology study; OR, odds ratio.

^a^ R^2^ equal to 0.389 (corresponding to 193 SNPs) was applied for calculations in this table. ^b^ OR of cancer per one unit increase in log-odds of T2DM.

## Figure S1: Flowchart showing the confirmation of prevalent diabetes cases

Abbreviations: DM, diabetes mellitus.

Those participants who gave a ‘YES’ response to the question ‘Has a doctor EVER told you that you had diabetes?’ were counted into ‘self-reported diabetes’. At the same time, those gave a ‘NO’ answer to the previous question, but satisfy one of the following criteria were regarded as ‘screen-detected diabetes’: (1) a random plasma glucose level of 126 mg/dL or greater (≥7.0 mmol/L) with time since last ate food of 8 hours or longer; (2) a random plasma glucose level of 200 mg/dL or greater (≥11.1 mmol/L) with time since last ate of less than 8 hours; (3) a fasting plasma glucose level of 126 mg/dL or greater (≥7.0 mmol/L).

## Figure S2: Illustration of the longitudinal matching design in the present study

**(A)** The schema of sequential allocations of participants to the exposed or unexposed group. The relevant time scale for matching was the calendar time (*d*). Follow-up for outcomes (time scale *t*) began after the assigned index date *d_x_*. The possible maximum follow-up time for every participant was the time lag between *d_a_* and *d_x_*, where *d_a_* was the administrative censoring date. **(B)** Illustration of two hypothetical participants in a matched pair, where participant ***a*** was the initially exposed individual and participant ***b*** the matched unexposed. A *per-protocol* analysis is presented here**,** where participant ***b*** was followed up as unexposed (indicated by green bar) from the assigned index date to a new diagnosis of T2DM in this matched pair, and in another stratum, as an exposed individual with new follow-up since T2DM diagnosis (indicated by red bar).

## Figure S3: Illustration of the study design in Mendelian Randomisation

Abbreviations: SNPs, single nucleotide polymorphisms; MR, Mendelian Randomisation; T2DM, type 2 diabetes mellitus; BBJ-CRC, Biobank Japan colorectal cancer cohort; CKB, China Kadoorie Biobank; JaPAN, Japan pancreatic cancer research; NCC, National Cancer Centre; BBJ-PaC, Biobank Japan pancreatic cancer cohort; KoGES, Korean Genome and Epidemiology Study; IVW, inverse-variance weighted; MR-PRESSO, MR Pleiotropy RESidual Sum and Outlier methods.

## Figure S4: Flowchart for identifying eligible individuals for matching at baseline

Abbreviations: DM, diabetes mellitus; BMI, body mass index; M, men; W, women.

## Figure S5: Age-standardised incidence rate of T2DM in men and women across the ten study regions

T2DM risks (per 100,000 person-years) were standardised by baseline age (<40/40–49/50–59/60–69/≥70 years) using the total diabetes-free population at baseline in CKB as the standard. The size of each box is proportional to the number of new-onset T2DM cases in each group and error bars indicate the 95% confidence interval estimated by assuming a Poisson distribution.

## Figure S6: Distribution of age at T2DM diagnosis among participants eligible for matching as exposed individuals

**(A)** The histogram shows the distribution of age (years) at T2DM diagnosis for male participants who were eligible for matching. The grey dashed line indicates the 95th percentile (78.7 years). **(B)** The histogram shows the distribution of age (years) at T2DM diagnosis for female participants who were eligible for matching. The grey dashed line indicates the 95th percentile (78.2 years).

## Figure S7: Distribution of baseline body mass index among participants eligible for matching as exposed individuals

**(A)** The histogram shows the distribution of baseline BMI (kg/m^2^) for male participants who were eligible for matching as exposed individuals. The grey dashed line indicates the 95th percentile (31.1 kg/m^2^). **(B)** The histogram shows the distribution of baseline BMI (kg/m^2^) for female participants who were eligible for matching as exposed individuals. The grey dashed line indicates the 95th percentile (32.1 kg/m^2^).

## Figure S8: Schoenfeld residuals for T2DM status from a standard Cox model with constant coefficients

**(A)** Men. Schoenfeld residuals are plotted against follow‑up time (years) for the unadjusted Cox model with a constant coefficient for T2DM status. The solid line represents the fitted trend, and the dashed lines indicate the 95% confidence interval. The plot shows non‑proportionality, with higher residuals in the first year. **(B)** Women. Schoenfeld residuals are plotted against follow‑up time (years) for the unadjusted Cox model with a constant coefficient for T2DM status. The solid line represents the fitted trend, and the dashed lines indicate the 95% confidence interval. The pattern is similar to that in men, with elevated residuals early after the index date.

## Figure S9: Cumulative incidence function for total first primary cancers and competing non-cancer deaths

**(A)** Men. Cumulative incidence curves for total first primary cancer (solid lines) and non‑cancer death as a competing event (dashed lines) are shown for the exposed (new‑onset T2DM) and matched unexposed groups over 60 months of follow‑up. **(B)** Women. Cumulative incidence curves for total first primary cancer (solid lines) and non‑cancer death as a competing event (dashed lines) are shown for the exposed (new‑onset T2DM) and matched unexposed groups over 60 months of follow‑up.

## Figure S10: Univariable Mendelian Randomisation for the associations of T2DM with major site-specific cancers in East Asians

Abbreviations: T2DM, type 2 diabetes mellitus; BMI, body mass index; BBJ-CRC, Biobank Japan colorectal cancer cohort; CKB, China Kadoorie Biobank; JaPAN, Japan pancreatic cancer research; NCC, national cancer centre; BBJ-PaC, Biobank Japan pancreatic cancer cohort; KoGES, Korean genome and epidemiology study; OR, odds ratio; SNPs, single nucleotide polymorphisms.

^a^ Estimates indicating OR of cancer per doubling in odds of T2DM.

## Figure S11: Forest plot of SNP-specific MR estimates for the association between genetically predicted pancreatic cancer and risk of T2DM

Abbreviations: T2DM, type 2 diabetes mellitus; OR, odds ratio; SNP, single nucleotide polymorphism; IVW, inverse-variance weighted.

Each point represents the causal estimate for an individual SNP, with horizontal lines showing the 95% CIs. The “All – Inverse variance weighted” estimate represents the combined causal effect from the IVW method.

^a^ Estimates are presented as OR of T2DM per doubling in pancreatic cancer odds.

## References

1.	Bray F, Laversanne M, Sung H, Ferlay J, Siegel RL, Soerjomataram I, Jemal A. Global cancer statistics 2022: GLOBOCAN estimates of incidence and mortality worldwide for 36 cancers in 185 countries. *CA Cancer J Clin* 2024;74:229–63.

2.	Smith K, Deutsch AJ, McGrail C, Kim H, Hsu S, Huerta-Chagoya A, Mandla R, Schroeder PH, Westerman KE, Szczerbinski L, Majarian TD, Kaur V, et al. Multi-ancestry polygenic mechanisms of type 2 diabetes. *Nat Med* 2024;30:1065–74.

3.	Burgess S. Sample size and power calculations in Mendelian randomization with a single instrumental variable and a binary outcome. *International Journal of Epidemiology* 2014;43:922–9.

4.	Suzuki K, Hatzikotoulas K, Southam L, Taylor HJ, Yin X, Lorenz KM, Mandla R, Huerta-Chagoya A, Melloni GEM, Kanoni S, Rayner NW, Bocher O, et al. Genetic drivers of heterogeneity in type 2 diabetes pathophysiology. *Nature* 2024;627:347–57.

5.	Wong HS-C, Tsai S-Y, Chu H-W, Lin M-R, Lin G-H, Tai Y-T, Shen C-Y, Chang W-C. Genome-wide association study identifies genetic risk loci for adiposity in a Taiwanese population. *PLoS Genet* 2022;18:e1009952.

6.	Tanikawa C, Kamatani Y, Takahashi A, Momozawa Y, Leveque K, Nagayama S, Mimori K, Mori M, Ishii H, Inazawa J, Yasuda J, Tsuboi A, et al. GWAS identifies two novel colorectal cancer loci at 16q24.1 and 20q13.12. *Carcinogenesis* 2018;39:652–60.

7.	Lin Y, Nakatochi M, Hosono Y, Ito H, Kamatani Y, Inoko A, Sakamoto H, Kinoshita F, Kobayashi Y, Ishii H, Ozaka M, Sasaki T, et al. Genome-wide association meta-analysis identifies GP2 gene risk variants for pancreatic cancer. *Nat Commun* 2020;11:3175.

8.	Nam K, Kim J, Lee S. Genome-wide study on 72,298 individuals in Korean biobank data for 76 traits. *Cell Genomics [Internet]* 2022 [cited 2025 Jan 21];2. Available from: https://www.cell.com/cell-genomics/abstract/S2666-979X(22)00138-0
